# Soya saponins and prebiotics alter intestinal functions in Ballan wrasse (*Labrus bergylta*)

**DOI:** 10.1017/S000711452200383X

**Published:** 2023-09-14

**Authors:** Weiwen Zhou, Kai K. Lie, Elvis Chikwati, Katerina Kousoulaki, Ingrid Lein, Øystein Sæle, Åshild Krogdahl, Trond M. Kortner

**Affiliations:** 1 Department of Paraclinical Sciences, Faculty of Veterinary Medicine, Norwegian University of Life Sciences (NMBU), Ås, Norway; 2 Feed and Nutrition, Institute of Marine Research, Bergen, Norway; 3 Nofima AS, Bergen, Norway; 4 Nofima AS, Sunndalsøra, Norway

**Keywords:** Cleaner fish, Ballan wrasse, Gut health, Antinutrients, Prebiotics

## Abstract

A 5-week feeding trial was conducted in the cleaner fish Ballan wrasse (*Labrus bergylta*) for a better understanding of the basic biology of the intestinal functions and health in this stomach less species. During the trial, Ballan wrasse was fed either a reference diet, the reference diet supplemented with (i) a commercial prebiotic (Aquate™ SG, 0·4 %) expected to have beneficial effects, (ii) soya saponins (0·7 %) expected to induce inflammation or (iii) a combination of the prebiotics and the soya saponins to find a remedy for gut inflammation. Blood, intestinal tissue and gut content from four consecutive intestinal segments (IN1 – IN4) were collected. No significant differences in fish growth were observed between the four dietary groups. Saponin supplementation, both alone and in combination with prebiotics, increased weight index of IN2 and IN3 and decreased blood plasma glucose, cholesterol and total protein. Dry matter of intestinal content and activity of digestive enzymes were not affected by diet. Histomorphological analyses revealed a progressing inflammation with increased infiltration by immune cells particularly into the distal parts of the intestine in fish fed diets with saponins, both alone and in combination with prebiotics. Gene expression profiles obtained by RNA sequencing and quantitative PCR mirrored the histological and biochemical changes induced by the saponin load. The study demonstrated that Ballan wrasse gut health and digestive function may be markedly affected by feed ingredients containing antinutrients.

The Ballan wrasse (*Labrus bergylta*) has, during the recent years, become a domesticated fish species due to its ability to feed on sea lice living on the surface of Atlantic salmon (*Salmo salar*)^(1–3)^. Sea lice infestation has become the most important challenge for Atlantic salmon production in Norway and neighbouring countries^(1,4)^ as a result of increased lice resistance against delousing chemicals^(5)^. Ballan wrasse is considered as an effective therapeutic and preventive biological control agent against salmon lice^(6,7)^. Although wild caught fish are still the main source of Ballan wrasse used in salmon production, farmed Ballan wrasse is increasingly being used. However, the challenges in this production are many, and several knowledge gaps must be filled. The present study addressed questions whether nutrients sources can secure or compromise gut health in Ballan wrasse. The observation of severe symptoms of gut inflammation in an earlier survey of gut health in cultivated Ballan wrasse^(8)^ demonstrated the urgency for better understanding of the general structure and physiology of the digestive tract of this species and relationships between diet composition and digestive and immune functions in the intestine. The present study aimed to strengthen knowledge on such aspects of the short and agastric digestive tract of the Ballan wrasse^(9,10)^.

Soya saponins and a commercial prebiotic were chosen as stimuli to trigger immune responses in gut. In studies with Atlantic salmon, soybean meal has been documented to induce gut inflammation^(11–13)^ due to its content of saponins. Saponins seem to impair intercellular junctions and increase membrane permeability when they present in intestine^(14)^. The increase in permeability, and the resulting increase in influx into the tissue of antigens and other alien components, results in triggering of several immune functions, such as production of cytokines^(15,16)^ and migration of lymphocytes into the tissue^(17)^.

Supplementing fish with prebiotics has been reported to enhance immune status of intestine, improve gut structure and disease resistance with possible direct or indirect modulatory effects on gut immune responses^(18)^. Therefore, prebiotics have become useful tools for exploration of mechanisms of gut functions and health in fish. The prebiotic used in the current study is a commercial product (Aquate, Alltech) containing a cell wall extract from baker’s yeast (*Saccharomyces cerevisiae*) and dried algae. It is claimed by the producer to maintain gastrointestinal integrity and stability, and to improve nutrient digestibility and intestinal health (see https://www.knowde.com/stores/alltech/products/aquate/).

The saponins and the prebiotic were included in the diets alone or in combination, as tools for the study of mechanisms underlying gut function and health in Ballan wrasse. We hypothesised that soya saponins in diet would impair intestinal immunity, while the prebiotic would trigger immune reactions in the intestine and counteract the saponin effects when present in diet together.

## Materials and methods

### Experimental animals, diets and sampling

The experimental procedure of the present study composed of a 5-week feeding trial with four diets, i.e. a reference diet (Ref) and three experimental diets made by supplementation with a prebiotic (Pre) expected to improve gut health and function at a level suggested by the producer, soya saponins (Sap) at a level found in soybean meal and found to induce inflammation in Atlantic salmon^(15,19)^ or a mixture of the two (P + S), which might indicate whether gut inflammation can be prevented by functional ingredients. Together observations made on fish fed these four diets were expected to fulfil the aims of this study, i.e. to be able to improve diet composition and thereby health and gut function of the fish by strengthening knowledge of the general structure and physiology of the digestive tract of Ballan wrasse and relationships between diet composition and digestive and immune functions in the intestine.

The feeding trial followed the Norwegian animal welfare act guidelines, in accordance with the Animal Welfare Act of 20 December 1974, amended 19 June 2009. The facility at Sunndalsøra, Nofima (division 60) got a permission granted by the Norwegian Food Safety Authority (FOTS ID 8060) to run the experiment. The decision was made on the basis of Regulation 18 June 2015 on the use of animals in experiments, §§ 6, 7, 9, 10 and 11. The 3R principles were used as a foundation for the design of the experiment, i.e. to ‘Reduce, Refine, Replace’, which means to minimise the use of experimental animals to a minimum, to keep the animals in such a way that the burden on the animals is as small as possible, and when possible, animal experiments are replaced by alternative methods. As effects of antinutrients and prebiotics on digestive functions and gut health, indicated by biochemical, histological and molecular responses, were the main goals of the present work, and effects of growth were not in focus, two tanks per treatment were considered sufficient for the generation of information on tank effects. The variables addressed have shown negligible tank effects from our previous work with antinutrient in diets for Atlantic salmon. Moreover, 5 weeks feeding was considered sufficient, as development of effects of saponins in Atlantic salmon takes only about 2 weeks to reach full inflammation pictures^(20,21)^.

The fish used for the trial had been reared for 1 year in a commercial farm (MOWI, Bergen, Norway) before transfer to the Nofima AS land-based research facility in Sunndalsøra, Norway. Upon arrival, the fish were distributed randomly to ten cylindrical flat-bottomed black tanks with a volume of 350 l, equipped with separate light sources and artificial kelp. The photoperiod was 24 h per day. A flow through system was used in the tank, with seawater pumped from the Sunndal fjord and filtered by drumfilters of approximately 20 µm. The water salinity was 32 ppt, and water temperature in tanks during the experiment was 14·5–17°C. All fish were individually tagged (passive-integrated transponder, weighed and length measured at the same time as they were tagged. Because of the sensitivity of Ballan wrasse to the change of feed and tank environment, fish were kept on the same diet before the start of the experiment. After 2-week rearing, the feed uptake remained low, so fish were reared for another 2 weeks in the experimental tanks. During these 4 weeks, the fish were fed the diet later referred to as the reference diet. At the start of the experiment, fish weight was 73·5 g, and the densities were adjusted to 16 kg/m^3^ per tank (80 fish).

One reference diet (Ref) containing high-quality marine raw materials was made (Table 1). The diet choice of ingredients and formulation was based on experience from previous Ballan wrasse feeding trials^(22,23)^. It contained 61 % protein, 16 % lipid, 7 % carbohydrate and 16 % ash (of dry matter). Three experimental diets were made by supplementing the Ref diet with either a commercial prebiotic (Aquate™ SG, 0·4 %, a yeast product containing *Saccharomyces cerevisiae* extract and algae, included at a level recommended by the producer Alltech) (Pre), soyasaponin (0·7 %) (Sap) or the combination of prebiotics and soyasaponin (P + S). Feed was produced by extrusion (2 mm pellet size) at Nofima’s Aquafeed Technology Center in Bergen, Norway. Feed was distributed randomly to duplicate groups of fish per diet for 24 h with 10 min feeding intervals, using automatic belt feeders for the duration of the experiment. Each belt feeder (one per tank) was loaded with feed equivalent to 80 g feed/kg fish per day. Before loading to each belt automat, the 80 g of feed was soaked in 10 ml of water for 4–5 min to soften the feed. Feed intake was not recorded.

**Table 1.** Basic feed formulation (%)¶¶

	Ref
Shrimp meal*	28·50
Cod meal*	24·83
Krill hydrolysate†	6·44
Reference fishmeal stick water‡	12·00
Squid meal*	11·00
Fish oil§	3·50
Krill oil||	2·50
Wheat¶	7·00
Vitamin mix**	2·65
Mineral mix**	5·00
Soya lecithin††	2·00
Additives‡‡	2·08
Yttrium oxide§§	0·05
Water	–7·55
Sum	100·00

* Provided by Seagarden AS, Norway.

† Antarctic krill (*Euphausia superba*) provided by RIMFROST AS, Norway.

‡ Provided by Pelagia AS, Norway.

§ Provided by Norsildmel AS, Norway.

|| Provided by Aker Biomarine Antarctic AS, Norway.

¶ Provided by Norgesmøllene, Norway.

** Supplied by Vilomix, Norway.

†† Provided by Denofa, Norway.

‡‡ Cholesterol from Grudlita, Lithuania; Choline chloride, Carophyll Pink (10%), Stay-C and Methionine from Vilomix, Norway; Lysine from Agrocorn, Denmark; and Taurine from VWR, Norway.

§§ Provided by Metal Rare Earth Limited, China.

¶¶ 0·4 %Aquate (provided by Alltech, Norway) and/or 0·7% soya saponins (purity ≥ 80%, provided by Shanxi Pioneer Biotech Co., Ltd., Xian, China) were added to make treatment diets (Pre, Sap and P + S).

The feeding trial ran for 5 weeks. At termination of the feeding trial, fish were randomly sampled from the tanks and fully anaesthetised and euthanised with 110 mg/l tricaine methane sulfonate (MS-222, Argent Chemical Laboratories). From six fish per tank, blood was sampled by venipuncture of the caudal vein. Blood was collected in vacutainers containing lithium heparin and stored on ice until centrifugation. Plasma was separated and immediately frozen in liquid nitrogen and stored at –80°C until analysis. After blood withdrawal, fish were killed by a blow to the head before organ sampling. Thereafter, fish were opened, and the viscera was removed from the abdominal cavity for dissection. The intestine was cleaned of mesenteric fat and divided into four segments (IN1–4) as previously described^(9)^. Each segment was opened longitudinally, and the gut content was collected, snap frozen in liquid nitrogen and stored at –80°C before further processing. From the same intestinal segments, tissue samples were taken for RNA extraction (submerged in RNAlater solution, incubated at 4°C for 24 h and stored at –20°C) and histomorphological evaluation (fixed in 4 % phosphate-buffered formaldehyde solution for 24 h and transferred to 70 % ethanol for storage).

### Blood plasma variables

Blood plasma was analysed for cholesterol, free fatty acids, total TAG, total protein and glucose at the Central Laboratory of the Faculty of Veterinary Medicine, Norwegian University of Life Sciences, Oslo, according to standard medical procedures. The instrument used was a Siemens Atellica CH fra Siemens Healthineers. The procedures are available in Supplementary files S1–S5.

### Histology

Formalin-fixed tissue samples were processed using standard histological techniques and stained with haematoxylin and eosin (H&E). Examination was conducted blindly and in randomised order. The following histological characteristics associated with inflammatory reactions in the intestinal mucosa were evaluated: length and fusion of mucosal folds, cellular infiltration, width of the lamina propria and submucosa, enterocyte vacuolisation, nucleus position within the enterocytes and the relative number of goblet cells. The morphological assessment was guided by our experience with salmonid intestinal histopathology^(24)^ as well as an examination of the histomorphology of the intestine in wild Ballan wrasse that we describe in Zhou et al^(25)^.

### Trypsin activity and total bile salts in the digesta

Trypsin activity and total bile salt levels were measured in pooled freeze-dried gastrointestinal contents from the four separate intestine segments (IN1–4). Trypsin activity was determined colorimetrically as described by Kakade and co-workers^(26)^ using the substrate benzoyl arginine p-nitroanilide (Sigma no. B-4875, Sigma Chemical Co., St. Louis, MO) and a curve derived from standardised bovine trypsin solution.

Bile salt levels were determined using the enzyme cycling amplification/Thio – NAD method (Inverness Medical, Cheshire, UK) in the ADVIA®1650 Chemistry System (Siemens Healthcare Diagnostics Inc.) at the Central Laboratory of the Faculty of Veterinary Medicine, Norwegian University of Life Sciences, Oslo.

### Brush border membrane enzyme activity

The activity of the brush border membrane enzymes leucine aminopeptidase (LAP) and maltase was measured in tissue homogenates from IN1, IN2 and IN4 as described previously^(12)^. Due to an analytical error, some data from IN3 were lost and are therefore not presented. Activities were calculated as mmol substrate hydrolysed per unit time in the whole tissue per kg body weight (enzymatic capacity). Protein was analysed using the Bio-Rad Protein Assay (Bio-Rad Laboratories, Munich, Germany).

### Gene expression

#### RNA sequencing

Gene expression profiling was conducted using samples from IN4, based on our previous finding that the distal intestine harbours the most active immune functions^(27)^, in line with what is the case also in Atlantic salmon that shows distinct structural changes in this section upon feeding with diets containing saponins^(15)^. Gene expression analyses were performed using *n* = 3 fish per tank (*n* = 6 fish per diet) fed the three experimental diets and the reference diet. Total RNA was extracted using a BioRobot® EZ1 and RNA Tissue Mini Kit (Qiagen, Hilden, Germany) including a DNase treatment step according to the manufacturer’s instructions. RNA quantity and quality were assessed using a NanoDrop ND-1000 UV–vis Spectrophotometer (NanoDrop Technologies, Wilmington, USA). All samples had 260/230 and 260/280 ratios above 2·0 and 2·2, respectively, indicating high RNA purity. Agilent 2100 Bioanalyzer and RNA 6000 Nano LabChip kit (Agilent Technologies, Palo Alto, USA) were used for assessment of RNA integrity for the individual samples. The average RNA integrity number of all samples was 7·8 ± 0·5. RNA sequencing was performed by the Norwegian Sequencing Centre (www.sequencing.uio.no). Sequencing libraries were prepared with an automated NeoPrep platform (Illumina) using 90 ng total RNA input to the TruSeq Stranded mRNA Library Prep Kit (Illumina) and standard Illumina adaptors included in the kit. The libraries were sequenced using HiSeq4000 according to manufacturer instructions, generating paired end libraries with an average library size of 14·2 ± 1·4 million reads prior to mapping and remaining sequencing adaptors were removed using Cutadapt^(28)^. Subsequent trimming and filtering were conducted using Sickle (https://github.com/najoshi/sickle) with a 40 bp minimum remaining sequence length, Sanger quality of 20 and no 5’ end trimming. The individual libraries were mapped to the Ballan wrasse genome (European Nucleotide Archive accession number: PRJEB13687, http://www.ebi.ac.UK/ena/data/view/PRJEB13687), using the short read aligner TopHat2^(29)^, and transcript abundances were estimated using the FeatureCounts^(30)^. Differentially expression of genes (DEG)were analysed using the DEseq2 package^(31)^, taking advantage of the 2 × 2 factorial design and enabelling analysis of combining effects between saponin and prebiotic. Features (genes) with row sums less than fifty were excluded prior to analysis of the results for differently expressed genes. RNAseq can be accessed through the Gene Expression Omnibus (GEO accession number GSE152475). An adjusted *P* value (*P*-adjust) of < 0·1 (Benjamini and Hochberg correction) was applied for further downstream analysis. Pathway and functional analysis of DEG was conducted using the ingenuity pathway analysis (IPA) software (IPA, Qiagen, Redwood City, CA, USA). Canonical pathway analysis, disease and biological function analysis and prediction of upstream regulators were conducted on the data set. While both canonical pathway analysis and biological function analysis predict affected biological events, the upstream regulator analysis identifies the cascade of upstream transcriptional regulators that can explain the observed gene expression changes. IPA results were filtered using *P* < 0·05 (Fisher’s exact test) and a Z-score > 2 or higher depending on the need for reducing the data set. While the p value filtering reflects the likelihood of a pathway/function being significantly affected by the treatment, the Z-score describes the likelihood of a directional association in our data set. Positive and negative Z-scores imply an increase or decrease in activity/activation of the enriched pathway, respectively.

#### Quantitative real-time PCR

In addition to RNA sequencing, quantitative real-time PCR (qPCR) was used as an independent gene expression profiling method to examine genes of interest in detail. Assays were carried out according to MIQE standards^(32)^ on separate RNA aliquots from all samples used for RNA sequencing. Complementary DNA synthesis, qPCR assays, primer design, optimisation and validation were carried out as described previously^(33)^. For reference gene selection, we searched for stably expressed genes in the RNA sequencing data set by employing the following filter strategy: base mean > 200, log2fold < 0·01 and > 0·01. Three putative reference genes (*ywhae*, *gapdh2* and *top2a*) were selected from the list, and *gapdh2* was ultimately used as the only reference gene according to its stability in different diet groups. Target gene expression was normalised according to Muller *et al.* ^(34)^. Details of qPCR assays are given in Table 2. In total, we profiled nineteen genes based on either significant differential expression as obtained with RNAseq data analysis or by selecting gene candidates for assessment of gut functions and health.

**Table 2.** Primers and related information for qPCR assays

Gene symbol	Gene function	5' – 3' primer sequence		Amplicon size (bp)	Annealing temperature (°C)	Efficiency	Gene bank accession no.
		Forward	Reverse				
*gapdh2*	Synthesises pyruvate from D-glyceraldehyde 3-phosphate	TATTTGTGTCCGTGTGCCCC	GCCTCCGTCCACTGATGAAT	129	62	1·99	XM_020633887·1
*ctra*	Protein digestion	AGCGTCCCAGTAGAGAAGGT	ACACTGGAGCTGAATCTGGC	110	60	2·00	XM_020657256
*slc23a1*	Vitamin C transport	CCCACTGAACACCTCACACA	AGACCAATCAGCAGCTCCAC	93	60	1·83	XM_020655303
*cd36*	Lipid transport	ACGGAGGGATAAAACGCACA	TATGCTGTGGTTCCAGGCTC	181	62	2·00	XM_020649455·1
*fabp2*	Lipid transport	TACAGCCTTGCGGATGGAAC	ATCCTCTTAGCCTCCACACCT	173	60	1·95	XM_020643842·1
*aqp8*	Water channel	TTGGCTCCTTTCCTTGTGGG	CCGAGAATGAGCCTGAGCAA	197	60	1·95	XM_020642545·1
*sqle*	Cholesterol biosynthesis	ACGAGAGATCAGCGACCAAC	CAGGTTCTGGAGCCACTGTT	117	62	1·94	XM_020635029
*cyp51a1*	Cholesterol biosynthesis	AAGGACTGCTGTTCCGATGG	CCTCTCCACAAAACCACCGA	113	60	1·79	XM_020648620
*il1b*	Pro-inflammatory cytokine	AAGGACGGTGATGAGGCAAC	GAAACCGAACCATGTCGCTG	94	57	1·87	XM_020651384·1
*il6*	Pro-inflammatory cytokine	GATCCTTGGTGCGACCGAT	GAAGGGCAGCTTTCTGGGAG	117	57	1·95	XM_020631726·1
*nlrc3*	Negative regulator of innate immune responses	GGACTTGTTCCTCCGCTTCTCC	CTGATTGGTCTGTGAGCCACTTC	88	62	1·98	XM_020659897·1
*lyz*	Antibacteria	CTTGGGACAGCGAGGAACAC	TCCATCGCCCATGTTGTAGG	140	62	1·96	XM_020660641
*cd40*	Activates antigen presenting cells	AGCAGTAAACCCGACTGAGG	GCTTTGGTCGTCCTCGTTCT	85	60	1·99	XM_020651338·1
*igm*	Humoral immunity	ATCTCTTGTGGAACAGGGCAC	CCTTGAAGTCAGCAAAACGCT	101	55	1·89	XM_020660315·1
*cd38*	Synthesis and hydrolysis of cyclic ADP-ribose and NADP	CCTGCACAGATGACGACCAA	TTGCACATGTTTGGCACGAG	81	62	2·00	XM_020633573
*mrc1*	Mediates endocytosis of glycoproteins by macrophages	TGTCCGGTTACATCCAAAGGA	TTATTACCCCCAGAACCTGTGA	185	57	2·00	XM_020635202·1
*pcna*	Cell proliferation	GCCAACAACACACAAAGGCT	TCGTCTTTCTGCGTCACTCC	106	62	1·88	XM_020647462·1
*cat*	Catalysis of hydrogen peroxide	TGTGAGCCGTTACAACAGTGC	TTGGATGAAGAGCTGGGCTCC	142	60	1·62	XM_020660768·1
*mmp13*	Tissue remodelling	TCTCGACGCCGCTTATGAAA	CACGCACGGGTTTATAGCCA	95	60	1·90	XM_020631204·1
*fcgbp*	Mucin forming	CAACTCTCCCTGTCTCTCCAG	GCTTCACAGAGGCAATTCTCC	126	62	2·00	XM_020655516·2

### Calculations

Thermal growth coefficient (TGC) was calculated as TGC = 1000 × (FBW^1/3^ – IBW^1/3^) × (ΣD°)^-1^, where IBW and FBW are the initial and final body weights (tank means), and ΣD° is the thermal sum (feeding days × average temperature in °C).

The specific growth rate (SGR) was calculated using the tank means for initial body weight (IBW) and final body weight (FBW) as follows: SGR = [(ln FBW – ln IBW) /number of days] × 100.

Condition Factor (CF) was calculated as CF = FBW⁄ FL^3^ *100, where FBW and FL are final body weight and final length.

Organosomatic indices were calculated as percentages of the weight of the organ in relation to body weight.

### Statistical analyses

Data were processed using R (version 3.5.2, 2018) in the integrated development environment Rstudio (version 1.1.463, 2018). Tank effect was firstly examined by point plot followed by comparison of mixed-effect models. As tank effect in this study was not significant for any of the biomarkers, individual fish was used as the statistical unit. Data were then diagnosed for normal distribution of residuals and homogeneity of variance according to Kozak and Piepho^(35)^. Data that violated either normality of distribution of residuals or homogeneity of variance were subject to Box–Cox transformation.

Two-way ANOVA was used for statistical evaluation with diet and gut sections as class variables. When the interaction of factors was significant, one-way ANOVA followed by Tukey’s test was applied to investigate effects of diets in individual gut segments. Kruskal–Wallis test was applied for those data that could not meet normal distribution of residuals, followed by Dunn’s multiple comparison test if significant. Differences were considered significant at *P* < 0·05.

Differences in histological scores for the various evaluated morphological characteristics were analysed using ordinal logistic regression. When score differences were only two levels, statistical significance was assessed using the Fisher’s exact test. *Post hoc* analysis for significant test results was conducted using the Chisq.post.hoc test (Fifer package in R). Differences were considered significant at *P* < 0·05.

## Results

In accordance with the goals of the present work, presentation of the results are organised in two subchapters. The first reports results regarding the general physiology of the intestine of the Ballan wrasse, the second effects of the prebiotic and the saponins, alone and in combination.

### General characteristics of the intestine

**Fig. 1.**
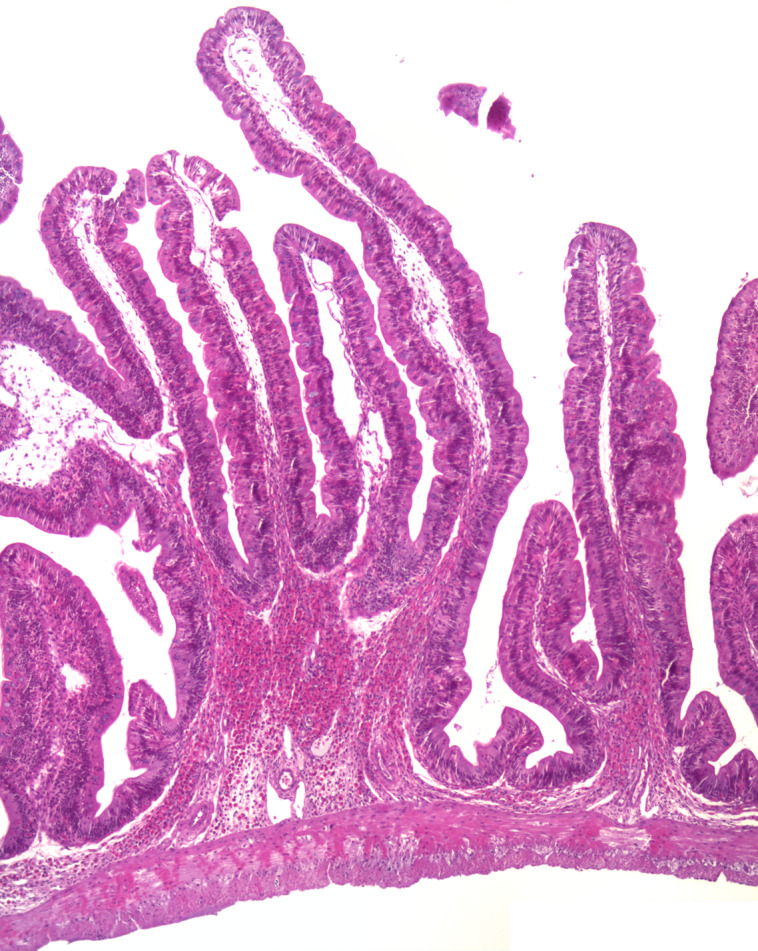
A representative image showing the bifurcation of intestinal mucosal fold.

**Fig. 2.**
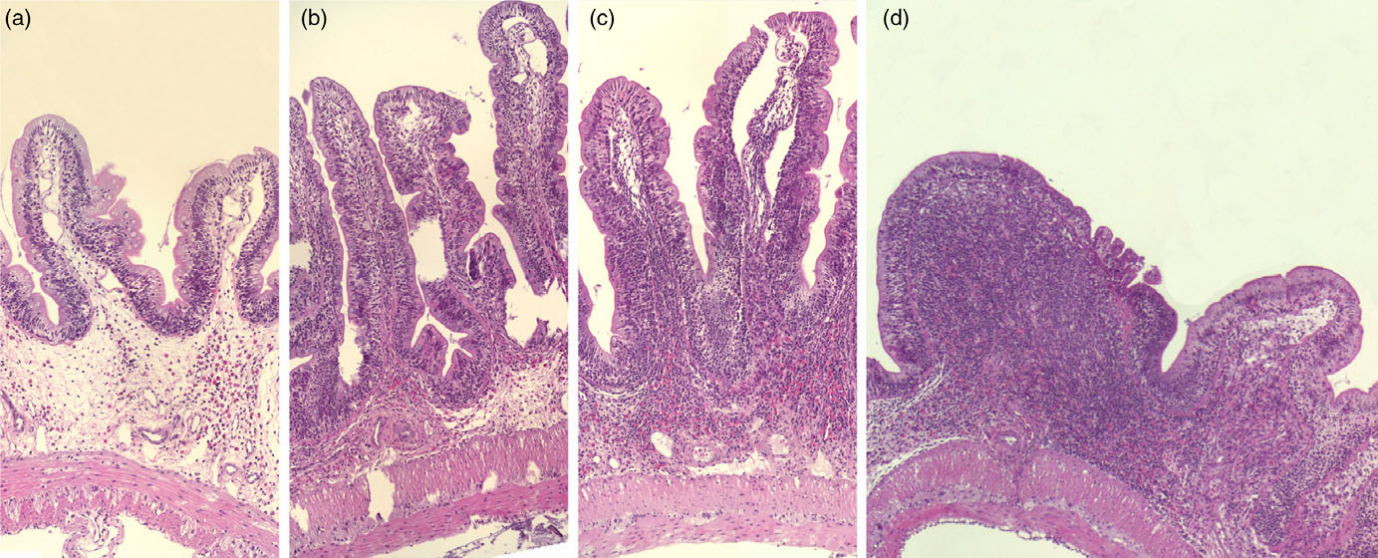
Representative images of the mucosa of Ballan wrasse intestine that were scored as normal, mild, moderate and marked (images a-d, respectively) for infiltration of lamina propria by inflammatory cells. Image d shows a notable increase in width of the lamina propria.

**Fig. 3.**
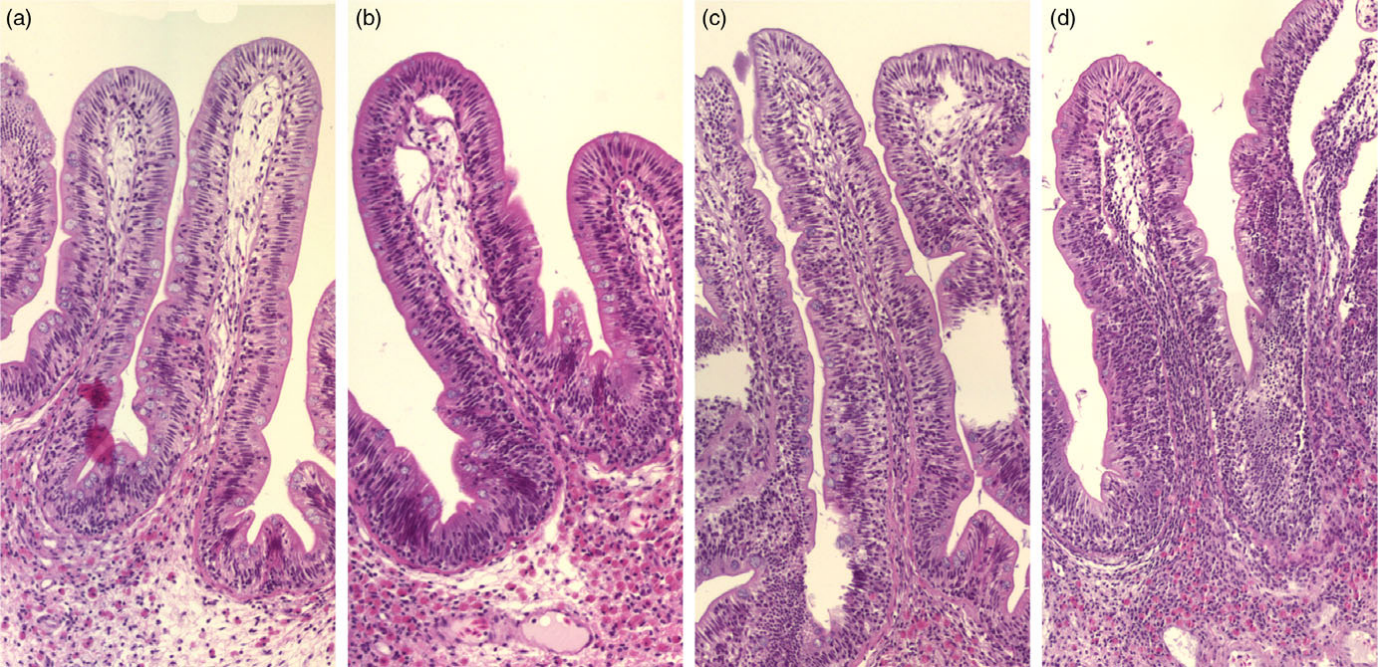
Representative images of the mucosal epithelium of the Ballan wrasse intestine that were scored as normal, mild, moderate, or marked for intraepithelial lymphocyte infiltration (images a-d, respectively).

The histological observations showed that both branched and unbranched mucosal folds appeared in the intestine of Ballan wrasse, and the branching occurred in all regions of the intestinal tract. The branching was mostly simple bifurcation (Fig. 1), and they were most extensive in the IN1 and IN4. The submucosa and lamina propria were wide, containing fibrous tissue and showed varying degrees of cellularity (Fig. 2(a)). The most common cell type in these compartments was the eosinophilic granular cells. A stratum compactum delimiting the submucosa from the muscular layers, observed in some other species, was absent in the Ballan wrasse. The Ballan wrasse intestinal mucosa showed a high degree of intraepithelial lymphocytes. The enterocytes seemed to lack extensive supranuclear vacuolisation (Fig. 3) normally found in the distal intestine of Atlantic salmon^(13)^.

Table 3 shows the two-way ANOVA results of variables including organosomatic indices, LAP and maltase capacity, dry matter, trypsin activity and bile salt level in digesta. The organosomatic indices, given as percent of body weight, of the intestinal segments were 0·37 ± 0·02, 0·35 ± 0·02, 0·23 ± 0·01 and 0·13 ± 0·01 for IN1, IN2, IN3 and IN4, respectively (mean ± S.E.M), showed a decreasing trend towards the distal segments.

**Table 3.** *P* values of the results based on two-way ANOVA

	*P* value			
Parameters	Model	Diet	Segment	Diet × Segment
OSI (% of body weight)	< 0·01	0·049	< 0·01	0·32
Maltase capacity (μmol/min/kg fish)	< 0·01	0·08	< 0·01	< 0·01
LAP capacity (mmol/h/kg fish)	< 0·01	0·04	0·06	0·046
DM (%)	0·48	0·09	0·64	0·22
Trypsin activity (U/g DM)	0·75	0·69	0·77	0·94
Bile salt level (mg/g DM)	< 0·01	0·09	0·04	0·03

OSI, organosomatic index; LAP leucine aminopeptidase; DM, dry matter.

**Fig. 4.**
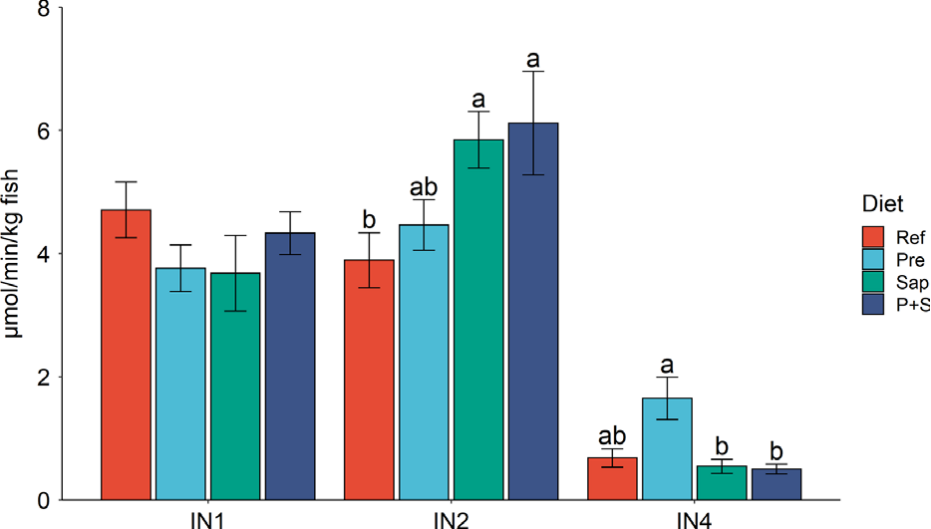
Maltase capacities in IN1, IN2 and IN4. Values are means with standard errors as error bars. Mean values with different letters are significantly different. *P* values derived from one-way ANOVA for diet effect were 0·30, less than 0·01 and less than 0·01 in IN1, IN2 and IN4, respectively. IN1, IN2 and IN4, intestinal regions 1, 2 and 4 in a proximo-distal axis.

**Fig. 5.**
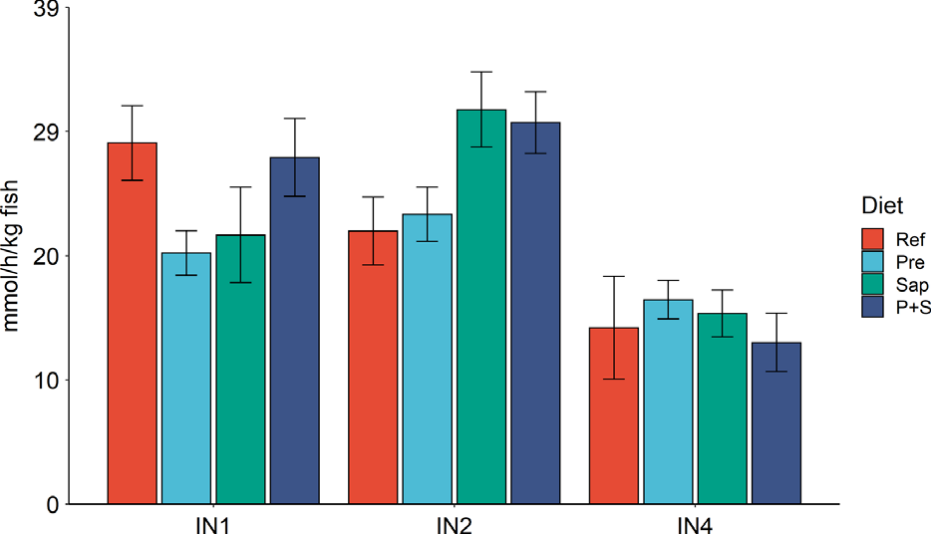
LAP capacities in IN1, IN2 and IN4. Values are means with standard errors as error bars. Mean values with different letters are significantly different. *P* values derived from one-way ANOVA for diet effect were 0·11, 0·22 and 0·39 in IN1, IN2 and IN4, respectively. IN1, IN2 and IN4, intestinal regions 1, 2 and 4 in a proximo-distal axis.

**Fig. 6.**
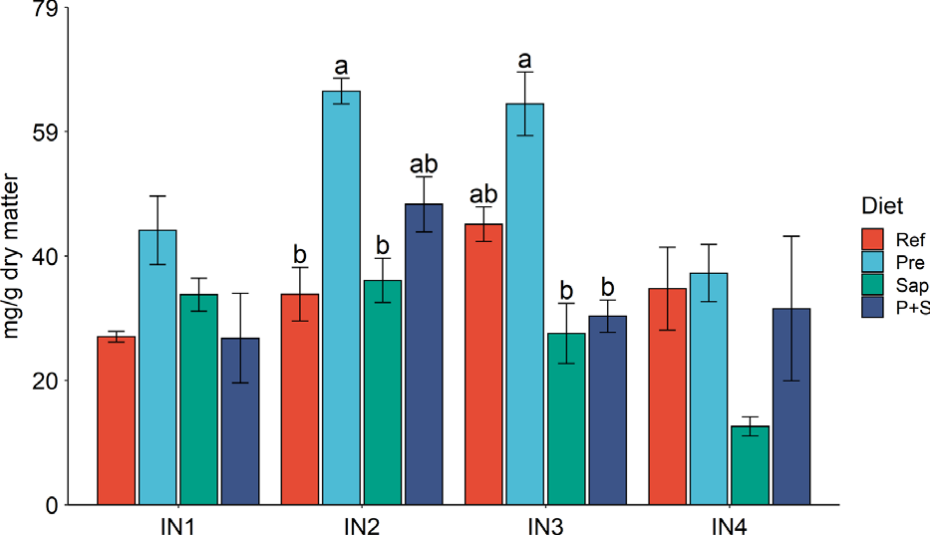
Digesta bile salt concentration in IN1 to IN4. Values are means with standard errors as error bars. Mean values with different letters are significantly different. *P* values derived from one-way ANOVA for diet effect were 0·16, 0·01, less than 0·01 and 0·20 from IN1 to IN4, respectively. IN1–4, intestinal regions 1–4 in a proximo-distal axis.

Trypsin activity, analysed in the intestinal chyme, LAP and maltase activity in homogenates of the mucosa of the measured segments demonstrated digestive capacity throughout the intestinal tract, with a decreasing trend towards the distal part of the intestine (Fig. 4 and Fig. 5). Bile salts were also detected in digesta collected from IN1 to IN4 with the highest level in IN2 (Fig. 6).

### Effects of saponins and prebiotics

#### Fish growth

Initial and final weight and length, and condition factor (CF), TGC, and SGR are presented in Table 4. There were no significant differences in fish growth performance between the treatments. Regarding the mortality, except that 2·7 % fish died due to an incidence with the oxygen supply in one tank, there was no fish died during the feeding trial.

**Table 4.** Initial and final weight, length, condition factor (CF), thermal growth coefficient (TGC) and specific growth rate (SGR)

	Ref	Sap	Pre	P + S	*P* value	Pooled sem
Initial weight (g)	72·4	73·8	70·7	72·9	0·19	0·83
Final weight (g)	89·7	92·5	87·5	93·9	0·56	1·67
Initial length (mm)	162·1	162·3	160·6	161·6	0·38	0·38
Final length (mm)	170·4	171·8	169·4	173·1	0·69	0·88
CF (g cm^-3^)	1·79	1·81	1·78	1·79	0·69	0·008
TGC (g^1/3^(°C day)^-1^)	0·29	0·28	0·26	0·32	0·78	0·013
SGR (% day^-1^)	0·32	0·31	0·29	0·35	0·79	0·014

#### Blood plasma biochemistry

Results for the blood plasma variables are presented in Table 5. Adding the prebiotic alone to the diet (Pre) reduced plasma glucose in fish compared with that of the Ref diet. Fish fed the Sap and the P + S diets showed similar decreasing trends. The Pre diet did not affect plasma cholesterol level, whereas Sap and P + S diets reduced cholesterol level. Plasma total protein level was unaffected by Pre, but showed a decreasing trend for the Sap diet, and a significant decrease in fish fed the P + S diets. Regarding plasma TAG level, none of the supplemented diets caused significant changes.

**Table 5.** Blood plasma variables*

	Ref	Sap	Pre	P + S	*P* value	Pooled sem
Glu (mmol l^-1^)	2·1^a^	1·5^ab^	1·4^b^	1·5^ab^	0·01	0·5
FFA (mmol l^-1^)	5·5	5·7	5·7	5·9	0·15	0·4
TG (mmol l^-1^)	11·3^ab^	11·2^b^	15·8^a^	12·2^ab^	0·01	3·8
Chol (mmol l^-1^)	6·4^a^	3·7^b^	6·3^a^	4·1^b^	< 0·01	1·2
Tprot (g l^-1^)	48^a^	40^ab^	48^a^	38^b^	< 0·01	8

* FFA, free fatty acid; Glu, glucose; Chol, cholesterol; Tprot, total protein. Values with different superscript letters within the same row denotes significant difference (*P* < 0·05).

#### Histomorphology of the intestine

**Fig. 7.**
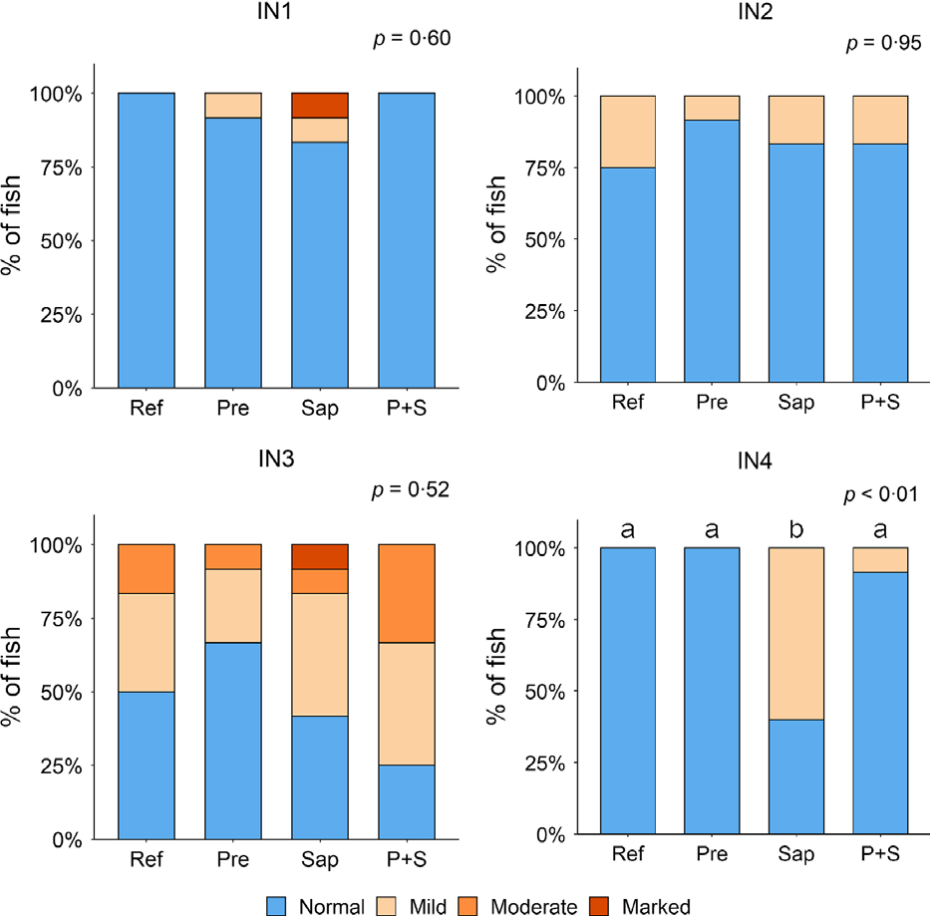
Diet effects on the cellularity of the lamina propria in the different regions of the Ballan wrasse intestine. LP, lamina propria. IN1–4, intestinal regions 1–4 in a proximo-distal axis.

**Fig. 8.**
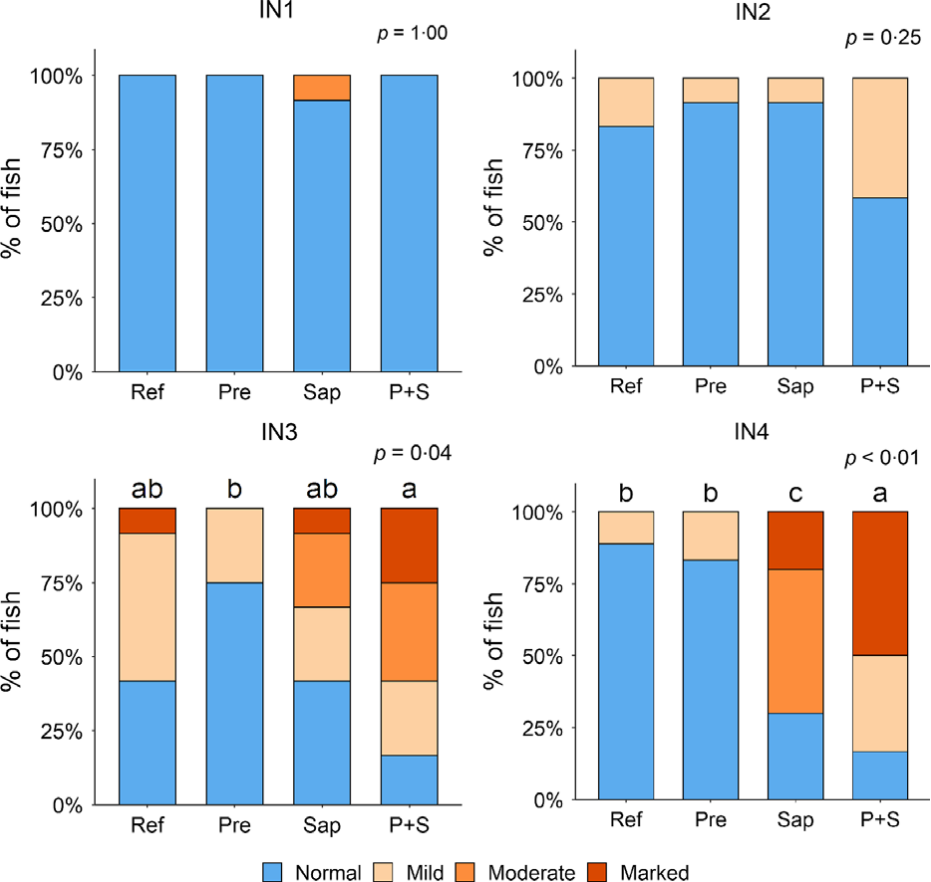
Diet effects on the intraepithelial lymphocyte infiltration in the different regions of the Ballan wrasse intestine. IEL, intraepithelial lymphocyte. IN1–4, intestinal regions 1–4 in a proximo-distal axis.

**Fig. 9.**
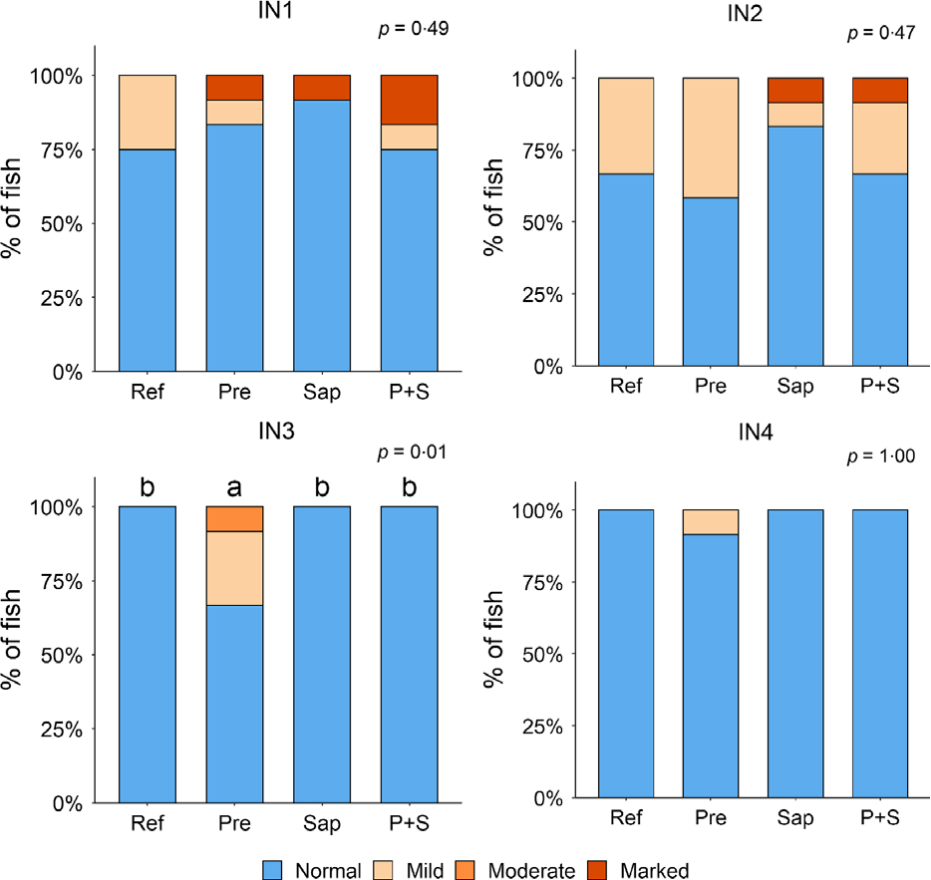
Diet effects on the numbers of intraepithelial rodlet cells and eosinophilic granular cells (EGCs) in the mucosal epithelium in the different regions of the Ballan wrasse intestine. IN1–4, intestinal regions 1–4 in a proximo-distal axis.

Adding the prebiotic alone (Pre) to the Ref diet did not alter the cellularity of the lamina propria (Fig. 7) nor the intraepithelial lymphocyte infiltration (Fig. 8), but significantly increased intraepithelial eosinophilic granular cells in IN3 (Fig. 9). Adding soya saponins alone (Sap) caused moderate to marked changes regarding intraepithelial lymphocyte infiltration in IN3 and IN4 (Fig. 8), indicating a progressive inflammatory process. However, cellularity of the lamina propria (Fig. 7) and the number of eosinophilic granular cells were not affected (Fig. 9). Combining the prebiotic and the saponin (P + S) did not eliminate the saponin effects, but rather increased immune cell infiltration in lamina propria in IN4 (Fig. 7) and intraepithelial lymphocyte infiltration in IN3 and IN4 (Fig. 8).

In some individuals, marked infiltration of eosinophilic granular cells and increased rodlet cell numbers in the mucosal epithelium were observed in IN1 and IN2. However, the changes did not show relationship to dietary treatments.

#### Activity of digestive enzymes in the brush border membrane

The results for maltase capacity along the intestine, observed for the sections IN1, 2 and 4, are presented in Fig. 4. No significant diet effects were observed in IN1 for any of the diets. In IN2 Pre did not affect maltase activity, whereas significant stimulating effects of Sap and P + S were observed. The results for IN4 showed a trend towards stimulating effects of Pre but showed no effect of Sap or P + S. Regarding LAP capacity (Fig. 5), no significant changes in any of the intestinal segments were observed. However, the results showed a trend towards increased capacity for fish fed Sap and P + S, in parallel to the result observed for maltase.

#### Bile salt concentration, dry matter and trypsin activity in intestinal content

The Pre diet, compared to the Ref diet, elevated chyme bile salt levels significantly in IN2 and showed the same trend for IN1 and IN3 (Fig. 6). Neither the Sap nor the P + S diet caused significant effects on bile salt concentration in any of the intestinal segments. This indicates that the effect of the prebiotic was eliminated when combined with the saponins.

For dry matter and trypsin activity in the intestinal content, no diet effects were observed (Table 3). The trypsin activities averaged 181, 110, 110 and 131U/mg dry matter, whereas digesta dry matter was 16, 16, 17 and 16 % in IN1 to IN4, respectively.

#### Gene expression in IN4

#### RNA sequencing

**Fig. 10.**
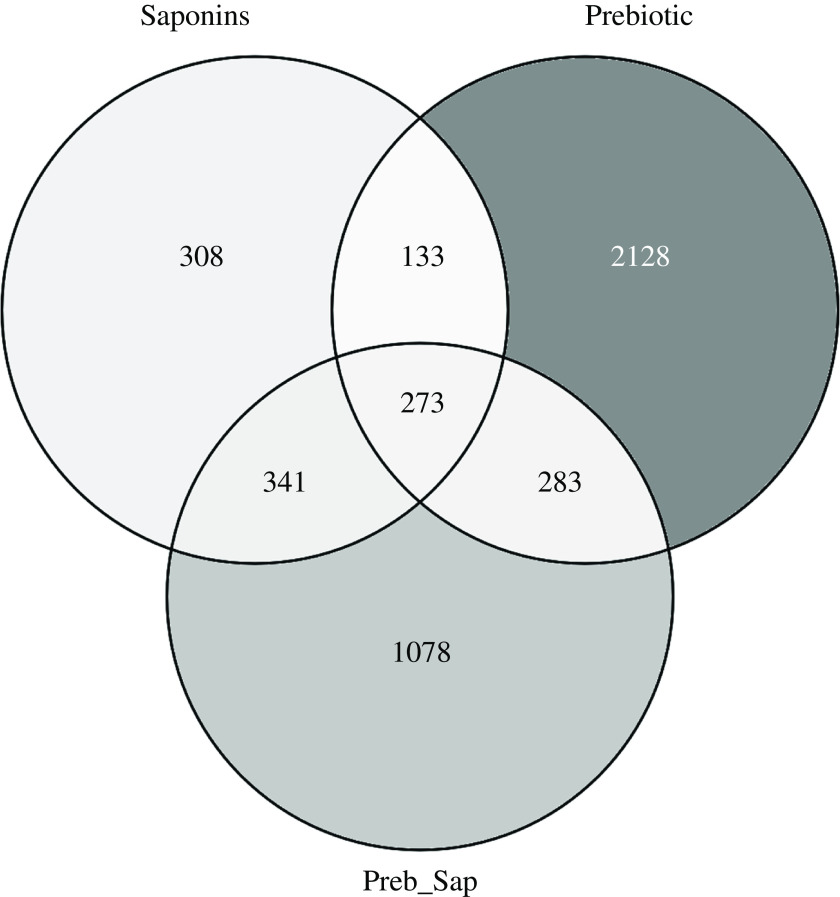
Venn diagram showing the number of differentially expressed genes affected by either saponin, prebiotic or their combination. Among them, 308, 2128 and 1078 genes were exclusively affected by saponin, prebiotic and combination treatments, respectively; 133 genes were influenced by both saponin and prebiotic treatments; 341 genes were influenced by both saponin and combination treatments; 283 genes were influenced by both prebiotic and combination treatments and 273 genes were influenced by all treatments. Venn diagram was created by using venny 2·1·0.

As illustrated in Fig. 10, a total number of 4544 genes showed effect of diet on their expression, as compared to the expression in fish fed the Ref diet. Among the treatments, fish fed the Pre diet demonstrated effect on the greatest number of genes, 2817 of which 2128 were unique for this treatment. Fish fed the Sap diet showed the lowest number: 1055 of which 308 were unique, and those fed the P + S diet showed effects on an intermediate number of genes:1975, of which 1078 were unique for this treatment. Among the DEGs, 273 were common for all the three supplementations, 133 for Sap and Pre, 341 for Sap and P + S, 283 for Pre and P + S. The complete list of DEGs is shown in supplemental table S6.

The ten most upregulated genes (log2 fold change > 2) for fish fed the Pre diet relatively diverse in their biological function such as *histone h2a* in gene regulation, *mtrf1l* in translation termination, *l1td1* in stem cell function, *mks1* in cell surface structure maintenance, *jam3* in regulation of tight junctions, *igkv4–1* in antigen binding, *ly75* in antigen processing, *itih3* in handling of oxidative stress, *me2* in mitochondrial energy metabolism and *hkdc* in glucose metabolism. For the Sap treatment, in contrast, most of the top upregulated genes were involved in lymphocyte function and signalling as a response to infestation, such as *neurl3*, involved in protein degradation, the pro-inflammatory chemokines *ccl11* and *ccl4*, *pnp* in purine metabolism*, mov10b* in RNA binding and the pro-inflammatory *il8r1.* A similar picture was observed for P + S for which *neurl3, ccl11* and *ccl4* were among the top abundant genes.

The top ten most down-regulated genes for fish fed the Pre diet belonged to diverse functional categories, such as *amdhd* in histidine metabolism, *blm* involved in DNA damage response and *ccer2* in microtubule bundling. Several of the most suppressed genes in fish fed Pre diet pointed towards cell development, differentiation and turnover, including *anxa4*, *ccnb*, *daam2* and *cdh2*. A more uniform picture of genes responsible for nutrient digestion, transport and metabolism was evident among the most down-regulated genes in fish fed Sap and the P + S combination. In particular, reduced transcript levels of genes involved in fatty acid uptake and metabolism (*cd36*, *fabp2*, *acsl5*, *nr1d2*, *acsl5* and *asah2*), lipoprotein synthesis (*apoeb*, *dgat2*) and sterol metabolism (*abcg8*, *cyp26a1*) were observed for fish fed Sap or P + S. Also, amino acid (*slc1a4*), peptide (*slc15a1*/*pept1*), starch (*mgam*) and vitamin A (*rbp2*) transporters were reduced by the Sap or the P + S.

Functional analyses of DEGs conducted by IPA software analyses revealed that several processes were affected by the individual dietary supplements (Pre and Sap) and that there was a significant interaction between prebiotics and saponin (online Supplementary figures S7 – S9). Following hierarchical clustering analysis of ‘Canonical pathways’ (Z-score < 2), ‘Disease and function’ (Z-score < 2,5) and ‘Upstream regulators’ (Z-score < 6), the Sap group and the P + S combination group of DEG clustered together in all three analysis (online Supplementary Fig. S10) suggesting that the expression fingerprint of the P + S was more similar to the Sap fed fish than to the Pre fed fish.

**Fig. 11.**
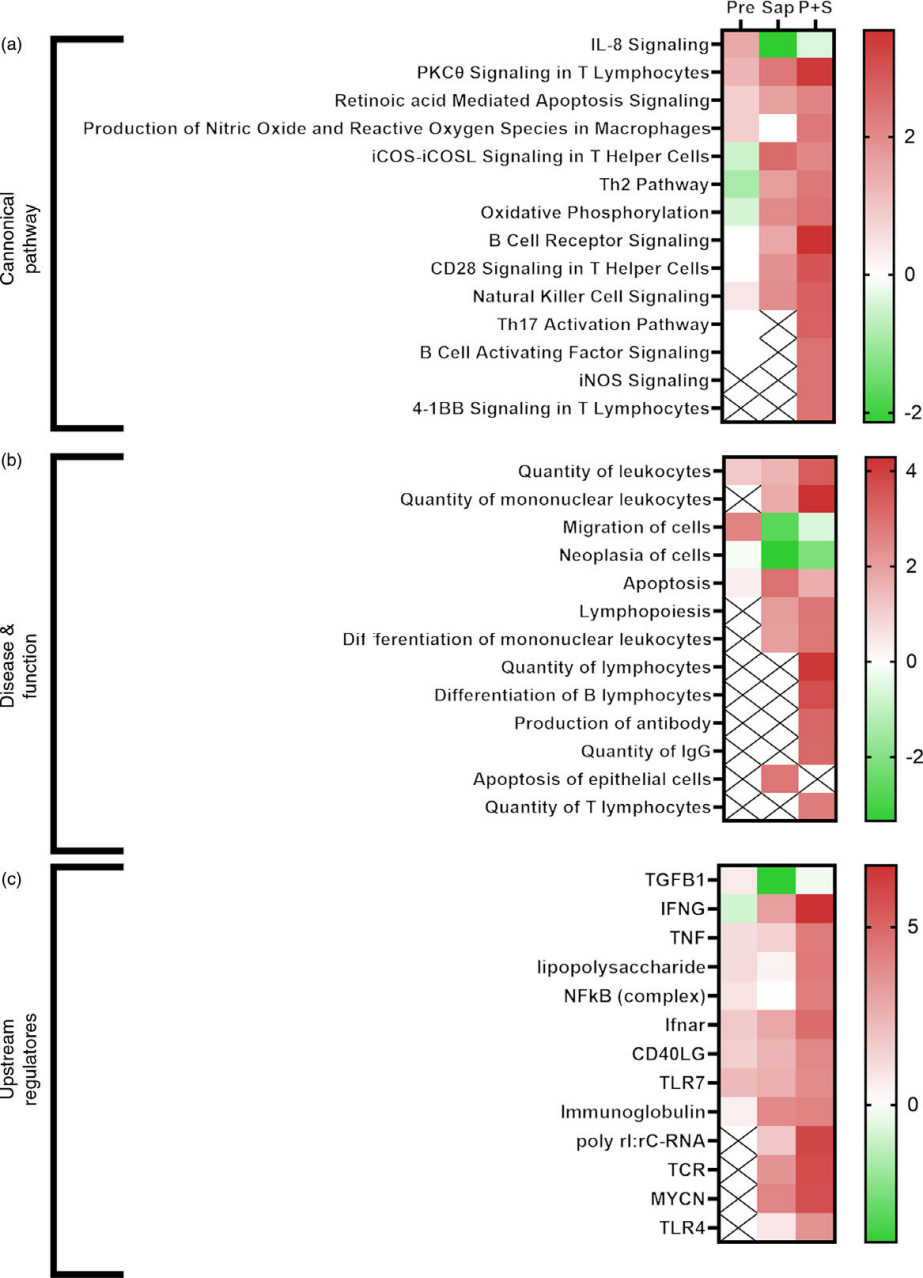
Comparative ingenuity pathway analysis showing A) significant canonical pathways (Z-score > 2), B) disease and function (Z-score > 2·5) and C) predicted upstream regulators (Z-score > 4) related to immune function in Ballan wrasse (*Labrus bergylta*) as a result of adding either saponins, prebiotics or saponin + prebiotic in the feed. All analysis were filtered using p < 0·05 in addition to Z-score filtering. Non-significant terms are marked as ‘X’.

Selected IPA terms related to immune function are presented in Fig. 11. Fish fed Pre showed significant induction of several immune pathways, including IL-8 signalling, reactive oxygen species production and T-cell function, that pointed towards upstream immune regulators such as TNF, lipopolysaccharide, NFkB and Toll-like receptor 7. For fish fed sap, a large number of immune pathways were significantly induced, including B- and T-cell development, signalling and apoptosis. Correspondingly, a number of upstream regulators related to these pathways also showed significant induction after Sap treatment, including interferon gamma, TNF, TLR7, immunoglobulins and T-cell receptor. Interestingly, the P + S combination seemed to potentiate the effect of Sap alone, showing strong induction of most of the same immune pathways related to B-cell, T-cell and macrophage function. A range of inflammatory and immunological molecules were predicted to be upstream regulators affecting the transcriptome of the P + S fed fish, with IFNG being the predicted molecule with the highest z score.

**Fig. 12.**
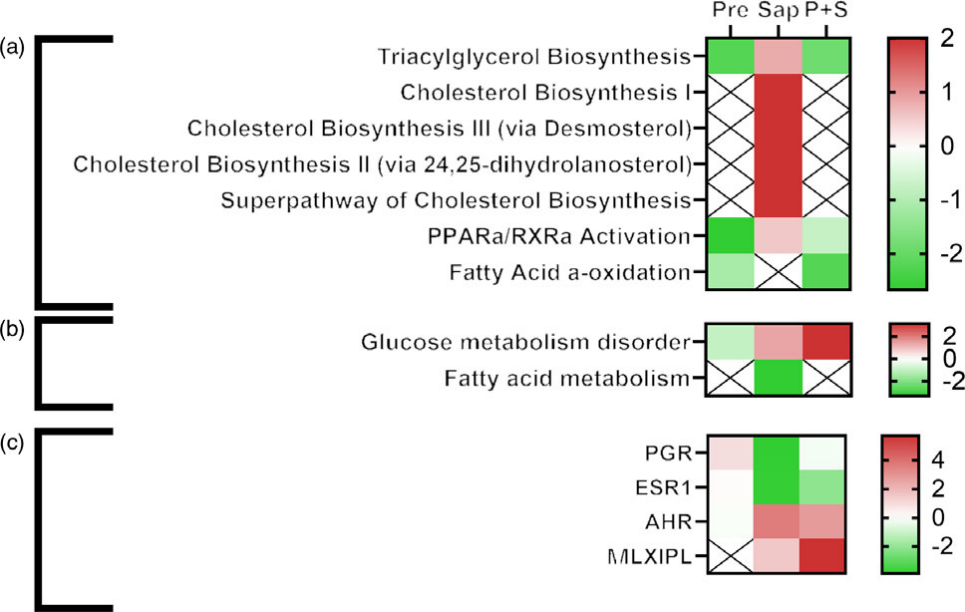
Comparative ingenuity pathway analysis showing A) significant canonical pathways (Z-score > 2), B) disease and function (Z-score > 2·5) and C) predicted upstream regulators (Z-score > 4) related to lipid metabolism in Ballan wrasse (*Labrus bergylta*) as a result of adding either saponins, prebiotics or saponin + prebiotic in the feed. All analysis were filtered using p < 0·05 in addition to Z-score filtering. Non-significant terms are marked as ‘X’.

In addition to the effects observed on the immune function terms, many IPA terms related to lipid metabolism were significantly affected by diet (Fig. 12). In particular, cholesterol biosynthesis was not affected by Pre, but strongly induced by Sap, whereas the combination of P + S seemed to alleviate the effect of saponins on cholesterol synthesis. TAG biosynthesis and fatty acid alpha oxidation were negatively affected by Pre and by the combination P + S. Several upstream regulators involved in lipid and sterol metabolism were also affected by the Sap and P + S treatments, such as progesterone, estrogen (ESR1) and aryl hydrocarbon receptors, and the carbohydrate-responsive element-binding protein encoded by MLXIPL.

#### Quantitative real-time PCR

**Fig. 13.**
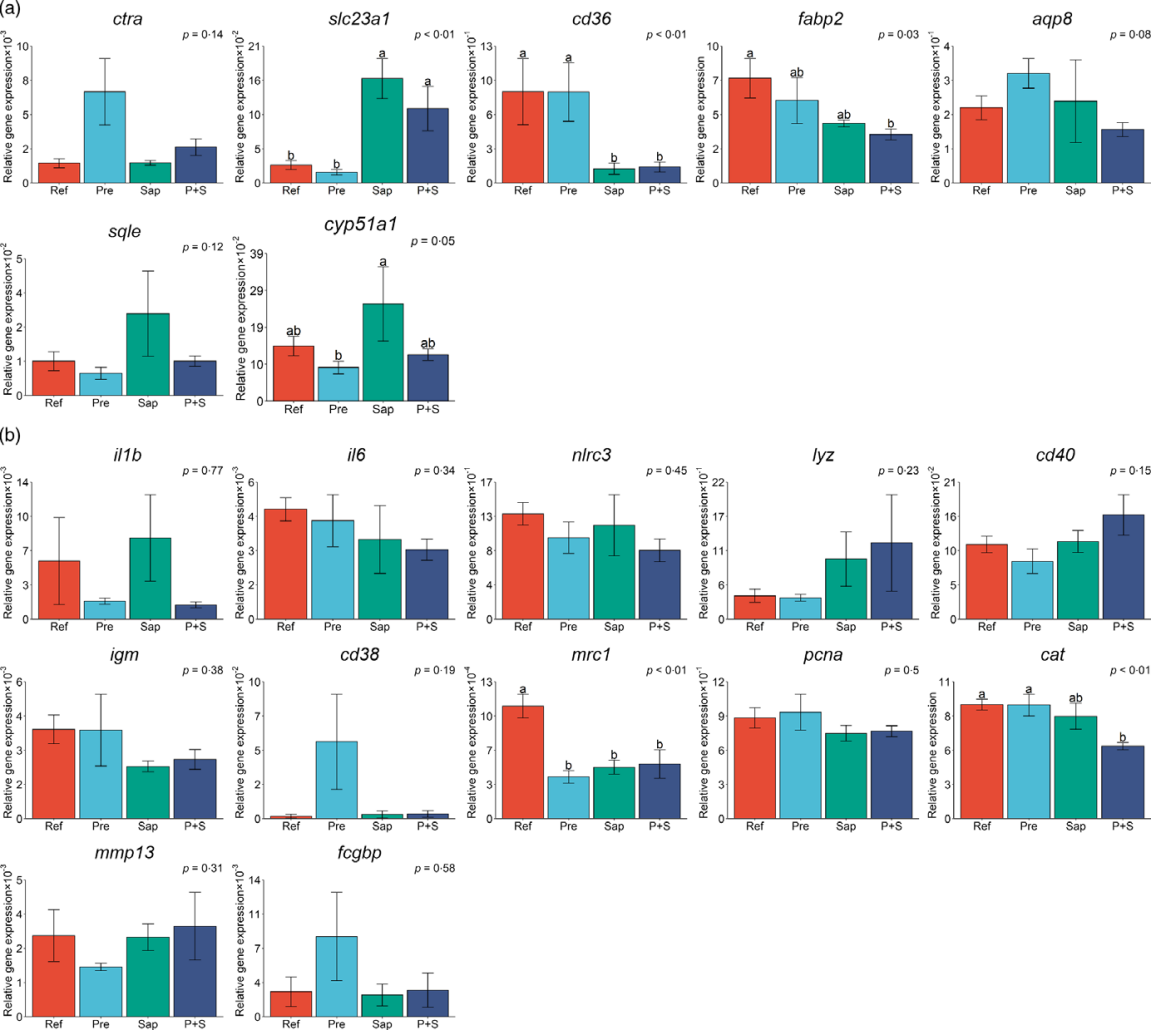
Distal intestine gene expression of A: chymotrypsin A (*ctra*), vitamin C transporter (*slc23a1*), cluster of differentiation 36 (*cd36*), fatty acid binding protein (*fabp2*), aquaporin 8 (*aqp8*), squalene epoxidase (*sqle*) and lanosterol 14α-demethylase (*cpy51a1*). B: interleukin 1 beta (*il1b*), interleukin 6 (*il6*), NOD-like receptor family CARD domain containing 3 (*nlrc3*), lysozyme (*lyz*), cluster of differentiation 40 (*cd40*), immunoglobulin M (*igm*), cluster of differentiation 38 (*cd38*), mannose receptor C-type 1 (*mrc1*), proliferating cell nuclear antigen (*pcna*), catalase (*cat*), matrix metallopeptidase 13 (*mmp13*), IgGFc-binding protein (*fcgbp*). Values are means with standard errors as error bars. Mean values with different letters are significantly different. *P* values derived from statistical analysis are given.

Results from the qPCR analyses are illustrated in Fig. 13(a) and (b), sorted in two panels according to their roles in the gut. Panel A includes genes related to digestive processes, i.e. protein digestion (*ctra*), nutrient absorption (*slc23a1*, *cd36*, *fabp2* and *aqp8*) and sterol metabolism (*sqle* and *cyp51a1*), and panel B includes genes related to immune regulation (*il1b*, *il6*, *nlrc3*, *lyz*, *cd40*, *igm*, *cd38* and *mrc1*), cell proliferation (*pcna*), oxidative stress (*cat*), cell remodeling (*mmp13*) and mucin formation (*fcgbp*).

Regarding the genes related to the digestive processes (13A), fish fed the Pre diet showed expression not significantly different from fish fed the Ref diet, although the results seemed to indicate a trend towards upregulation of the *ctra* gene coding for the principal precursor of the pancreatic proteases. In fish fed the Sap and P + S diets upregulation of was indicated for *slc23a1*, the vitamin C transporter, downregulation of one of the fatty acids transporters, *cd36*. Expression of *fabp2*, another fatty acid transporter, was downregulated only by the P + S diet. Fish fed the Sap diet exhibited a trend towards increased expression of genes involved in sterol metabolism (*sqle* and *cyp51a1*). The expression level of *mrc1*, a receptor mediating the endocytosis of glycoproteins by macrophages, was downregulated in fish fed all the three supplemented diets compared with the Ref diet.

Regarding the immune-related genes subject of qPCR analyses, most of them, including the mucin-forming-related gene, *fcgbp*, did not show significant effects of any of the supplemented diets. A trend towards elevation of expression level of *cd38,* an enzyme synthesises cyclic ADP-ribose and NADP, as well as *fcgbp*, was observed in fish fed the Pre diet. Whereas, fish fed the P + S diet showed lower expression of *cat*, the catalase.

## Discussion

### General characteristics of intestinal tract of the Ballan wrasse

The relative weights (organosomatic indices) of the intestinal sections of the fish, showing values decreasing along the intestine from 0·37 to 0·13 %, in sum comprising 1·1 % of body weight, were about half the values observed in our previous studies^(8,36)^. The reason for this may be the difference of fish weight, which was three times as high in the former studies as in the present, about 50–60g. On the other hand, trypsin activity and bile salt concentration of the chyme were at the same level. The same was true for the total capacity of brush border LAP and maltase. A difference between the present results and the previous was the capacities of the sections along the intestine^(8)^. The explanation for this difference was most likely differences in the positioning of the division between IN1 and IN2 of the intestine, which in the two first studies were more proximal than in the present^(8,36)^. The reason for this change was difficulties in defining the end of the first section, a bulb-like structure. In the latter study, the intestine was divided in four sections of similar length.

The results regarding the brush border enzyme capacity were quite similar in the present and the previous study^(8,36,37)^, i.e., the enzyme capacity in IN1 and IN2 was about 10 and 110 U/kg fish for maltase and LAP in the present study, about 10 and 50 U/kg fish in the previous ones. Also, trypsin activity and bile salt concentration in the intestinal content were similar. In the previous study, observations were not available for IN1. In IN2, the values in the present study were about 70 U and 40 mg/g dry matter, respectively, and in the previous study, the values were about 110 U and 40 mg/g dry matter.

The results for the distal most section indicated lower capacity than for the other sections, in the present as well as in the earlier studies^(8,36,37)^. The results confirm that IN1 - 3 have similar absorptive capacity and that reabsorption of enzymes and bile salts mostly take as the chyme passes through IN4. Efficient absorption of nutrients in IN1 - 3 seems to be secured by retrograde movements while the feed is present in the intestine^(38)^.

At the histological level, Ballan wrasse possesses wide submucosa and lamina propria. Thickening of submucosa and lamina propria usually is associated with unhealthy status in fish intestine^(12,24,39–42)^. Since wide submucosa and lamina propria commonly exist in Ballan wrasse, it may not be appropriated to involve them as a sign of unhealthy status of intestine. Instead, increase of cellularity that composed of leukocytes^(43)^ would be the more appropriate marker of unhealthy intestine in Ballan wrasse, which has also been observed in our previous study in wild Ballan wrasse^(25)^.

### Effects of prebiotics

The present study clearly indicated that supplementation with the prebiotics produced local effects in the gut on a molecular level, including modulation of innate and adaptive immunity. These responses were, however, not manifested in any clear histomorphological alternations in the gut. Previous studies indicate that prebiotics may strengthen disease resistance^(18)^. Potentially, the influenced genes are involved in pathogen and disease resistance, and therefore, changes were not triggered without infestations, in spite that in other fish species^(44,45)^ enhanced immunity was observed before pathogen challenge.

The prebiotics used in the present study is a product of yeast (*Saccharomyces cerevisiae*) extract and dried algae which contains functional ingredients such as mannan-oligosaccharides. The effects in fish would therefore be expected to be comparable to effects of mannan-oligosaccharides. In general, prebiotics appear to activate the innate immune system, directly or by enhancing the growth of commensal microbiota,^(46)^ but the effects seem to differ among different fish species. For instance, lysozyme expression was not altered in the present study, in line with observations in channel catfish (*Ictalurus punctatus*)^(47)^ and European sea bass (*Dicentrarchus labrax*)^(48)^ fed mannan-oligosaccharides supplemented diets. These results, however, differ from effects observed in rainbow trout (*Oncorhynchus mykiss*)^(49)^ and red drum (*Sciaenops ocellatus*)^(50)^ in which dietary mannan-oligosaccharides supplementation caused increase lysozyme activity. Adaptive immunity can also be influenced by prebiotics. For instance, lymphocyte responses, including both T- and B-cell responses, were enhanced in European sea bass (*D. labrax*) fed concentrated mannan-oligosaccharides supplemented diet^(51)^. The lymphocyte responses in Ballan wrasse, however, were not widely influenced by the supplemented prebiotics.

### Effects of soya saponins

The lowered plasma level of cholesterol and the trend towards lowered concentration of bile salts in the content of IN4 observed in fish fed Sap and P + S supports results from studies conducted with other fish species^(15,52,53)^. In the current study, saponin supplementation seemed to increase cholesterol synthesis in enterocytes, as indicated by the upregulation of *cyp51a1* and an elevating trend of *sqle* in fish fed Sap. These results agree with observations in Atlantic salmon showing increased synthesis of cholesterol and bile salts in the liver by feeding with diets containing soybean meal^(54)^. The intestine plays important roles in cholesterol homoeostasis through absorption and excretion. In addition, the enterocytes have the capacity to synthesise cholesterol. Enhancing cholesterol synthesis in enterocyte is likely a manner to counteract the lowering effect of saponins on plasma cholesterol level also in Ballan wrasse. An experiment with mice supports this assumption by showing upregulation of the expression of *Srebp2* gene, a regulator of sterol and fatty acid synthesis, and genes related to cholesterol synthesis, including *sqle* and *cyp51a1* ^(55)^, as well as increased plasma cholesterol.

The reduced expression in IN4 of genes involved in fatty acid uptake and metabolism, such as *cd36* and *fabp2* in the Sap and P + S treatments and other related genes verified by IPA in the Sap treatment, is in line with results from studies in Atlantic salmon^(15,16)^ and gilthead sea bream^(52)^, suggesting that saponin inclusion also influences fatty acid transport in the distal most compartment, directly or indirectly. However, blood plasma levels of free fatty acids and triglycerides were not significantly affected, indicating that the reduced expression of *cd36* and *fabp2* did not reduce availability of fatty acids to the peripheral tissues of the fish. The main reason for lack of effect on plasma lipid levels may be the fact that most lipids are absorbed in the more proximal compartments^(37)^. Similarly, in Atlantic salmon, it has been observed that dietary inclusion of soya saponin may reduce expression of *fabp2* and reduce apparent digestibility of lipid in the distal intestinal segment, but, as in the present study, plasma levels of free fatty acids and TAG remained unchanged^(15)^.

It is well established that soya saponins induce intestinal inflammation in some fish species, and the most typical signs in these are mobilisation of the immune apparatus, loss of digestive functions and major histological alterations^(13,15,56)^. The present results indicate that similar processes take place in the distal intestine of the Ballan wrasse. In Atlantic salmon, the affected genes are in particular related to T and B cell regulation^(21)^. In the present study, all the top ten genes upregulated by Sap are involved in immune functions such as lymphocyte functions and signalling, and pathways related to immunity like ‘T_h_2 pathway’ and ‘CD28 signalling in T helper cells’ were also induced. We therefore conclude that soya saponins induce similar responses in Ballan wrasse as in Atlantic salmon.

The histological changes observed in the present Ballan wrasse study indicated intraepithelial infiltration of lymphocytes. The infiltrating lymphocytes were most likely T cells as in the intestine of higher vertebrates, and intraepithelial lymphocytes were identified as diverse T cells^(57)^. The involvement of T lymphocytes is suggested to be a strategy for fighting the consequences of soya saponins as this has been commonly observed in Atlantic salmon^(20,21,58)^, which may be triggered by the increase in mucosal permeability by saponin, allowing entrance of alien compounds^(59)^. The present results indicate that the increased exposure to feed and microbial components from the intestine induced a wide range of very complex reactions with pro- and anti-inflammatory changes occurring simultaneously in the distal intestine. For instance, the downregulation of gene *mrc1* may be a reflection of anti-inflammation because it is considered a marker of macrophage in intestine^(60)^ and is found to be reduced also in gilthead seabream displaying anti-inflammatory status^(61)^. The inhibition of IL-8 signalling pathway was likely an indication of anti-inflammation processes since IL-8 participates in the proinflammatory signalling cascade^(62)^. The presence of anti-inflammatory changes may be a reaction for protecting the fish from negative effects of the induced inflammatory responses.

### Combination effects of saponin and prebiotics

The fact that the gene expression profile for P + S clustered with that of Sap indicates that the modulatory effects of prebiotics were overridden by the challenging effects of the Sap. The responses to P + S differed from the responses to Pre in particular regarding intraepithelial lymphocyte infiltration. They were more similar to responses to Sap with enhanced lymphocyte responses. Based on the gene expression results, the type of lymphocytes particularly involved in the P + S treatment can be suggested. For instance, T helper cell 17 (T_h_17) was particularly activated. It differentiates from CD4 + T cells together with regulatory T cells (T_reg_). It is demonstrated to participate in pathogen clearance and the balance of T_h_17 and T_reg_ influence autoimmunity^(63,64)^, and these may also be affected in the Ballan wrasse. Besides this, the type of B cells seems not to be IgM expressing, considering that *igm* expression showed no significant effect, but rather a decreasing trend. Instead, it might be IgT or IgD expressing B lymphocytes which, in earlier studies, have been found to be expressed in IN4 in Ballan wrasse^(65)^.

However, present information regarding combination effects of prebiotics and soya saponins in fish intestine are not sufficient to conclude regarding the mechanisms underlying the combined effects of the two substances. Considering the properties of the prebiotics, it is highly likely that effects on gut microbiota were important factors. However, one study with Nile tilapia has indicated that prebiotics and saponins do not necessarily alter the composition of gut microbiota^(66)^.

Altogether the results of the present study indicate that the use of prebiotics should be done with care. Interaction with antinutrient effects, such as effects of saponins, might eliminate their potential beneficial effects and possibly potentiate challenging effects of the antinutrients.

### Conclusions

In the present study, the Ballan wrasse responded to dietary soya saponins in a similar way as Atlantic salmon. Observed responses included decreased cholesterol levels in blood, and clear alterations in the histomorphology and gene expression of the distal intestine indicative of a progressing inflammation with increased intraepithelial lymphocyte infiltration. The study also suggested that dietary supplementation of prebiotics had modulatory, potentially beneficial, effects on gut health and function. However, interaction with plant antinutrients such as saponins may prevent these effects.

## Supplementary material

For supplementary material accompanying this paper visit https://doi.org/10.1017/S000711452200383X. click here to view supplementary material
