# Supplementary material for: Soya saponins and prebiotics alter intestinal functions in Ballan wrasse (Labrus bergylta)
Source: Br J Nutr. 2023 Jan 12;130(5):765–82. doi: 10.1017/S000711452200383X (PMC10404481; doi:10.1017/S000711452200383X)
Supplement: Supplementary file 1 [file S000711452200383Xsup001.zip › S000711452200383Xsup001.docx]

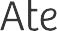

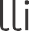

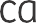

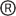

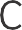


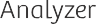
Cholesterol_2 (Chol_2)


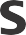

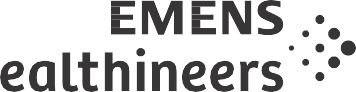


| **Current Revision and Date**a | Rev. 04, 2020-11 | |
| --- | --- | --- |
| **Product Name** | Atellica CH Cholesterol_2 (Chol_2) 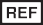 11097609 (8400 tests) | |
| **Abbreviated Product Name** | Atellica CH Chol_2 | |
| **Test Name/ID** | Chol_2 |  |
| **Systems** | Atellica CH Analyzer |  |
| **Materials Required but Not Provided** | Atellica CH CHEM CAL | 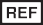 11099411 |
| **Specimen Types** | Serum, plasma (lithium heparin) |  |
| **Sample Volume** | 4 µL |  |
| **Measuring Interval** | 25–618 mg/dL (0.65–16.01 mmol/L) |  |

a A vertical bar in the page margin indicates technical content that differs from the previous version.


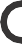

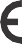


# Intended Use

The Atellica® CH Cholesterol_2 (Chol_2) assay is for *in vitro* diagnostic use in the quantitative determination of cholesterol in human serum and plasma (lithium heparin) using the Atellica® CH Analyzer. Such measurements are used in the diagnosis and treatment of disorders involving excess cholesterol in the blood and in lipid and lipoprotein metabolism disorders.

# Summary and Explanation

In the early 1980s, findings from the Coronary Primary Prevention Trial first demonstrated that lowering of a subject’s plasma cholesterol level reduces the incidence of coronary heart disease. Also, cholesterol is associated with a variety of disorders that result from hyperlipidemia and dyslipoproteinemia.

The Atellica CH Cholesterol_2 (Chol_2) assay is based on an enzymatic method using cholesterol esterase and cholesterol oxidase conversion followed by a Trinder endpoint.1-5

# Principles of the Procedure

The cholesterol esters are hydrolyzed by cholesterol esterase to cholesterol and free fatty acids. The cholesterol is converted to cholest-4-en-3-one by cholesterol oxidase in the presence of oxygen to form hydrogen peroxide. A colored complex is formed from hydrogen peroxide, 4-aminoantipyrine and phenol under the catalytic influence of peroxidase. The absorbance of the complex is measured as an endpoint reaction at 505/694 nm.

## Reaction Equation

Cholesterol Esterase

Cholesterol Ester + H2O Cholesterol + Fatty Acid

Cholesterol Oxidase

Cholesterol + O_2_ Cholest-4-en-3-one + H_2_O_2_

Peroxidase

H_2_O_2_ + Phenol + 4-Aminoantipyrine Quinoneimine Dye + 2H_2_O

# Reagents

| **Material Description Storage Stabilitya** |
| --- |
| **Atellica CH Chol_2** Unopened at 2–8°C Until expiration date on product  **Pack 1 (P1)** Onboard per well 26 days  Well 1 (W1)  Reagent 1 (R1)  19.4 mL  4-aminoantipyrine (1.25 mmol/L);  phenol (30.0 mmol/L);  peroxidase (horseradish) (≥ 2.5 U/mL); cholesterol esterase (Pseudomonas) (≥ 1.0 U/mL); cholesterol oxidase (microbial) (≥ 0.5 U/mL); sodium azide (0.09%)  Well 2 (W2)  Reagent 1 (R1)  19.4 mL  4-aminoantipyrine (1.25 mmol/L);  phenol (30.0 mmol/L);  peroxidase (horseradish) (≥ 2.5 U/mL); cholesterol esterase (Pseudomonas) (≥ 1.0 U/mL); cholesterol oxidase (microbial) (≥ 0.5 U/mL); sodium azide (0.09%) |

a Refer to [*Storage and Stability*](#_bookmark0)

## Warnings and Precautions

For *in vitro* diagnostic use. For Professional Use.

#### CAUTION

Federal (USA) law restricts this device to sale by or on the order of a licensed healthcare professional.

Safety data sheets (SDS) available on [siemens.com/healthineers](http://siemens.com/healthineers).

#### H319


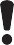


**P280, P337+P313**

**Warning!**

Causes serious eye irritation.

Wear protective gloves/protective clothing/eye protection/face protection. If eye irritation persists: Get medical advice/attention.

**Contains:** Alcohols, C12-14-secondary, ethoxylated (Atellica CH Chol_2 P1)

Contains sodium azide as a preservative. Sodium azide can react with copper or lead plumbing to form explosive metal azides. On disposal, flush reagents with a large volume of water to prevent buildup of azides. Disposal into drain systems must be in compliance with prevailing regulatory requirements.

Dispose of hazardous or biologically contaminated materials according to the practices of your institution. Discard all materials in a safe and acceptable manner and in compliance with prevailing regulatory requirements.

**Note** For information about reagent preparation, refer to [*Preparing the Reagents*](#_bookmark2) in the

[*Procedure*](#_bookmark1) section.

## Storage and Stability

Protect the product from light sources. Unopened reagents are stable until the expiration date on the product when stored at 2–8°C.

Do not use products beyond the expiration date printed on the product labeling.

## Onboard Stability

Reagents are stable onboard the system for 26 days per well. Discard reagents at the end of the onboard stability interval. Do not use products beyond the expiration date printed on the product labeling.

# Specimen Collection and Handling

Serum and plasma (lithium heparin) are the recommended sample types for this assay.

## Collecting the Specimen

- Observe universal precautions when collecting specimens. Handle all specimens as if they are capable of transmitting disease.6
- Follow recommended procedures for collection of diagnostic blood specimens by venipuncture.7
- Follow the instructions provided with your specimen collection device for use and processing.8
- Allow blood specimens to clot completely before centrifugation.9
- Keep tubes capped at all times.9

## Storing the Specimen

Specimens may be stored for up to 8 hours at 25°C or for up to 2 days at 2–8°C or for longer storage, specimens may be frozen at -20°C or colder.10,11

The handling and storage information provided here is based on data or references maintained by the manufacturer. It is the responsibility of the individual laboratory to use all available references and/or its own studies when establishing alternate stability criteria to meet specific needs.

## Transporting the Specimen

Package and label specimens for shipment in compliance with applicable federal and international regulations covering the transport of clinical specimens and etiological agents.

## Preparing the Samples

This assay requires 4 µL of sample for a single determination. This volume does not include the unusable volume in the sample container or the additional volume required when performing duplicates or other tests on the same sample. For information about determining the minimum required volume, refer to the online help.

**Note** Do not use specimens with apparent contamination.

Before placing samples on the system, ensure that samples are free of:

- Bubbles or foam.
- Fibrin or other particulate matter.

**Note** Remove particulates by centrifugation according to CLSI guidance and the collection device manufacturer’s recommendations.9

**Note** For a complete list of appropriate sample containers, refer to the online help.

# Procedure

## Materials Provided

The following materials are provided:

| 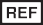 **Contents Number of Tests** |
| --- |
| 11097609 **Pack 1 (P1)** 4 x 2100  Well 1 (W1) 19.4 mL of Atellica CH Chol_2 Reagent 1 Well 2 (W2) 19.4 mL of Atellica CH Chol_2 Reagent 1 |

## Materials Required but Not Provided

The following materials are required to perform this assay, but are not provided:

| 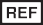 **Description** |
| --- |
| Atellica CH Analyzera |
| 11099411 Atellica CH CHEM CAL (calibrator) 12 x 3.0 mL calibrator  Calibrator lot-specific value sheet |
| Commercially available quality control materials |

a Additional system fluids are required to operate the system: Atellica CH Diluent, Atellica CH Wash, Atellica CH Conditioner, Atellica CH Cleaner, Atellica CH Reagent Probe Cleaner 1, Atellica CH Reagent Probe Cleaner 2, Atellica CH Reagent Probe Cleaner 4, Atellica CH Lamp Coolant, and Atellica CH Water Bath Additive. For system fluid instructions for use, refer to the Document Library.

## Assay Procedure

The system automatically performs the following steps:

1. For serum/plasma, dispenses 50 µL of primary sample and 200 µL of Atellica CH Diluent into a dilution cuvette.
2. Dispenses 16 µL of Reagent 1 and 64 µL of special reagent water into a reaction cuvette.
3. Dispenses 4 µL of pre-diluted sample into a reaction cuvette.
4. Mixes and incubates the mixture at 37°C.
5. Measures the absorbance after sample addition.
6. Reports results.

**Note** For information about special reagent water requirements, refer to the online help. Test Duration: 10 minutes

## Preparing the Reagents

All reagents are liquid and ready to use.

The Chol_2 reagent should be kept refrigerated at 2–8°C when not in use. The detergent in this reagent may appear cloudy if maintained at room temperature. If the reagent appears cloudy, restore it to a temperature of 2–8°C, and then mix the reagent by gently inverting the sealed reagent pack until the detergent precipitate dissolves. Once the reagent appears clear again, return the pack to cold storage in a refrigerator. Reagent performance is not affected once reagent cloudiness dissipates (or once reagent clarity is restored).

## Preparing the System

Ensure that the system has sufficient reagent packs loaded in the reagent compartment. For information about loading reagent packs, refer to the online help.

## Performing Calibration

For calibration of the Atellica CH Chol_2 assay, use Atellica CH CHEM CAL. Use the calibrators in accordance with the calibrator instructions for use.

### Calibration Frequency

Perform a calibration if one or more of the following conditions exist:

- - When changing lot numbers of primary reagent packs.
  - At the end of the lot calibration interval, for a specified lot of calibrated reagent on the system.
  - At the end of the pack calibration interval, for calibrated reagent packs on the system.
  - When indicated by quality control results.
  - After major maintenance or service, if indicated by quality control results.

At the end of the onboard stability interval, replace the reagent pack on the system with a new reagent pack. Recalibration is not required, unless the lot calibration interval is exceeded.

| **Stability Interval** | **Days** |
| --- | --- |
| Lot Calibration | 50 |
| Pack Calibration | 7 |
| Reagent Onboard Stability | 26 |

For information about lot calibration and pack calibration intervals, refer to the online help. Follow government regulations or accreditation requirements for calibration frequency.

Individual laboratory quality control programs and procedures may require more frequent calibration.

## Performing Quality Control

For quality control of the Atellica CH Chol_2 assay, use at least two levels (low and high) of the appropriate quality control material of known analyte concentration. Use the quality control material in accordance with the quality control instructions for use.

For the assigned values, refer to the lot‑specific value sheet provided. A satisfactory level of performance is achieved when the analyte values obtained are within the expected control range for the system or within your range, as determined by an appropriate internal laboratory quality control scheme. Follow your laboratory’s quality control procedures if the results obtained do not fall within the acceptable limits. For information about entering quality control definitions, refer to the online help.

Follow government regulations or accreditation requirements for quality control frequency. Individual laboratory quality control programs and procedures may require more frequent quality control testing.

### Taking Corrective Action

If the quality control results do not fall within the assigned values, do not report results. Perform corrective actions in accordance with established laboratory protocol. For suggested protocol, refer to the online help.

# Results

## Calculation of Results

The system determines the result using the calculation scheme described in the online help. The system reports results in mg/dL (common units) or mmol/L (SI units), depending on the units defined when setting up the assay.

Conversion formula: mg/dL x 0.0259 = mmol/L

For information about results outside the specified measuring interval, refer to [*Measuring*](#_bookmark3) [*Interval*](#_bookmark3).

## Interpretation of Results

Results of this assay should always be interpreted in conjunction with the patient’s medical history, clinical presentation, and other findings.

# Limitations

The Atellica CH Chol_2 assay is limited to the detection of cholesterol in human serum and plasma (lithium heparin).

Venipuncture should occur prior to N-Acetyl Cysteine (NAC) or Metamizole (Sulpyrine) administration due to the potential for falsely depressed results.

# Expected Values

## Reference Interval

A reference interval for healthy adults was established in accordance with CLSI Document EP28‑A3c and verified on the Atellica CH Analyzer.12

| **Reference Interval Group Specimen Type Common Units (SI Units)** |
| --- |
| Adults Serum and plasma13 Low-risk levels (desirable) < 200 mg/dL (< 5.18 mmol/L)  Moderate-risk levels (borderline) 200–239 mg/dL (5.18–6.19 mmol/L) High-risk levels ≥ 240 mg/dL (≥ 6.22 mmol/L) |

As with all *in vitro* diagnostic assays, each laboratory should determine its own reference interval for the diagnostic evaluation of patient results. Consider these values as guidance only.12

# Performance Characteristics

## Measuring Interval

The Atellica CH Chol_2 assay provides results from 25–618 mg/dL (0.65–16.01 mmol/L). The system flags all values that are outside the specified measuring interval.

## Extended Measuring Interval

An automatic repeat condition for this assay extends the measuring interval to 1236 mg/dL (32.01 mmol/L) for serum and plasma. You may configure the system to trigger an automatic repeat. Automatic repeat results will be flagged **Autorepeat**.

## Detection Capability

Detection capability was determined in accordance with CLSI Document EP17‑A2.14 The assay is designed to have a limit of blank (LoB) ≤ limit of detection (LoD), an LoD ≤ 10 mg/dL

(≤ 0.26 mmol/L), and a limit of quantitation (LoQ) ≤ 25 mg/dL (≤ 0.65 mmol/L).

The LoD corresponds to the lowest concentration of cholesterol that can be detected with a probability of 95%. The LoD for the Atellica CH Chol_2 assay is 2 mg/dL (0.04 mmol/L), and was determined using 120 determinations, with 60 blank and 60 low level replicates, and a LoB of 1 mg/dL (0.03 mmol/L).

The LoQ corresponds to the lowest amount of analyte in a sample that can be accurately quantitated with an inter-assay imprecision ≤ 20%. The LoQ of the Atellica CH Chol_2 assay is 25 mg/dL (0.65 mmol/L), and was determined using 4 patient samples that were assayed using 3 reagent lots, over a period of 3 days.

Assay results obtained at individual laboratories may vary from the data presented.

## Precision

Precision was determined in accordance with CLSI Document EP05‑A3.15 Samples were assayed on an Atellica CH Analyzer in duplicate in 2 runs per day for 20 days (N ≥ 80 for each sample). The following results were obtained:

| **Repeatability** | | | | | **Within-Lab Precision** | |
| --- | --- | --- | --- | --- | --- | --- |
| **Sample Type** | **N** | **Mean**  **mg/dL (mmol/L)** | **SDa**  **mg/dL (mmol/L)** | **CVb (%)** | **SD**  **mg/dL (mmol/L)** | **CV (%)** |
| Serum Pool | 80 | 115 (2.97) | 0.88 (0.02) | 0.8 | 1.49 (0.04) | 1.3 |
| Serum QC | 80 | 170 (4.41) | 0.99 (0.03) | 0.6 | 1.65 (0.04) | 1.0 |
| Serum QC | 80 | 276 (7.14) | 1.26 (0.03) | 0.5 | 2.12 (0.05) | 0.8 |
| Plasma Pool | 80 | 584 (15.13) | 3.1 (0.08) | 0.5 | 5.5 (0.14) | 0.9 |

a Standard deviation.

b Coefficient of variation.

Assay results obtained at individual laboratories may vary from the data presented.

## Assay Comparison

The Atellica CH Chol_2 assay is designed to have a correlation coefficient of ≥ 0.97 and a slope of 1.0 ± 0.05 compared to ADVIA® Chemistry 1800 Chol_2. Assay comparison was determined using the Deming linear regression model in accordance with CLSI Document EP09‑A3.16 The following results were obtained:

| **Specimen** | **Comparative Assay (x)** | **Regression Equation** | **Sample Interval** | **Na** | **rb** |
| --- | --- | --- | --- | --- | --- |
| Serum | ADVIA Chemistry 1800 Chol_2 | y = 0.97x + 1 mg/dL  (y = 0.97x + 0.03 mmol/L) | 40–618 mg/dL  (1.04–16.01 mmol/L) | 100 | 0.997 |

a Number of samples tested.

b Correlation coefficient.

The agreement of the assay may vary depending on the study design, comparative assay, and sample population. Assay results obtained at individual laboratories may vary from the data presented.

## Specimen Equivalency

Specimen equivalency was determined using the Deming linear regression model in accordance with CLSI Document EP09‑A3.16 The following results were obtained:

| **Specimen (y)** | **Reference Specimen (x)** | **Regression Equation** | **Sample Interval** | **Na** | **rb** |
| --- | --- | --- | --- | --- | --- |
| Lithium heparin plasma | Serum | y = 1.00x - 3 mg/dL  (y = 1.00x - 0.08 mmol/L) | 38–516 mg/dL  (0.98–13.36 mmol/L) | 50 | 0.999 |

a Number of samples tested.

b Correlation coefficient.

Agreement of the specimen types may vary depending on the study design and sample population used. Assay results obtained at individual laboratories may vary from the data presented.

## Interferences

### Hemolysis, Icterus, and Lipemia (HIL)

The Atellica CH Chol_2 assay is designed to have ≤ 10% interference from hemoglobin, bilirubin, and lipemia. Interfering substances at the levels indicated in the table below were tested in accordance with CLSI Document EP07‑A2 using the Atellica CH Chol_2 assay.17

Bias is the difference in the results between the control sample (does not contain the interferent) and the test sample (contains the interferent) expressed in percent. Bias > 10% is considered interference. Analyte results should not be corrected based on this bias.

| **Substance** | **Substance Test Concentration Common Units (SI Units)** | **Analyte Concentration mg/dL (mmol/L)** | **Percent Bias** |
| --- | --- | --- | --- |
| Hemoglobin | 500 mg/dL (0.311 mmol/L) | 162 (4.19) | 6 |
|  | 1000 mg/dL (0.622 mmol/L) | 244 (6.33) | 8 |
| Bilirubin, conjugated | 60 mg/dL (1026 µmol/L) | 126 (3.27) | 8 |
|  | 60 mg/dL (1026 µmol/L) | 223 (5.79) | -6 |
| Bilirubin, unconjugated | 20 mg/dL (342 µmol/L) | 166 (4.30) | 9 |
|  | 20 mg/dL (342 µmol/L) | 261 (6.76) | 2 |
| Lipemia (Intralipid®) | 1000 mg/dL (11.3 mmol/L) | 152 (3.94) | 2 |
|  | 1000 mg/dL (11.3 mmol/L) | 235 (6.10) | 2 |

Assay results obtained at individual laboratories may vary from the data presented.

### Non-Interfering Substances

The following substances do not interfere with the Atellica CH Chol_2 assay when present in serum and plasma (lithium heparin) at the concentrations indicated in the table below. Bias due to these substances is ≤ 10% at an analyte concentration of 208 mg/dL (5.39 mmol/L). These data were generated on the ADVIA Chemistry system with assay reaction conditions that are equivalent to those on the Atellica CH Analyzer.18

| **Substance** | **Substance Test Concentration Common Units (SI Units)** | **Analyte Concentration mg/dL (mmol/L)** | **Percent Bias** |
| --- | --- | --- | --- |
| Ascorbic Acid | 6 mg/dL (341 µmol/L) | 208 (5.39) | ≤ 10 |

Assay results obtained at individual laboratories may vary from the data presented.

## Standardization

The Atellica CH Chol_2 assay is traceable to the NCEP/CDC reference method, which uses SRM 909 reference materials from the National Institute of Standards and Technology (NIST).

Assigned values for calibrators are traceable to this standardization.18

# Technical Assistance

For customer support, contact your local technical support provider or distributor. [siemens.com/healthineers](http://siemens.com/healthineers)

# References

1. Burtis CA, Ashwood ER. *Tietz Fundamentals of Clinical Chemistry.* 5th ed. Philadelphia, PA: Saunders: 2001.
2. Richmond W. Preparation and properties of a cholesterol oxidase from *Nocardia sp.* and its application to the enzymatic assay of total cholesterol in serum. *Clin Chem.* 1973;19(12):1350–1356.
3. Roeschlau P, Bernt E, Gruber W. Enzymatic determination of total cholesterol in serum. *Z Klin Chem Klin Biochem.* 1974 May;12(5):226.
4. Allain CC, Poon LS, Chan CS, et al. Enzymatic determination of total serum cholesterol. *Clin Chem.* 1974 Apr;20(4):470-475.
5. Trinder P. Determination of glucose in blood using glucose oxidase with an alternative oxygen acceptor. *Ann Clin Biochem.* 1969;6:24-27.
6. Clinical and Laboratory Standards Institute. *Protection of Laboratory Workers from Occupationally Acquired Infections; Approved Guideline—Fourth Edition*. Wayne, PA: Clinical and Laboratory Standards Institute; 2014. CLSI Document M29‑A4.
7. Clinical and Laboratory Standards Institute. *Procedures for the Collection of Diagnostic Blood Specimens by Venipuncture; Approved Standard—Sixth Edition*. Wayne, PA: Clinical and Laboratory Standards Institute; 2007. CLSI Document GP41‑A6.
8. Clinical and Laboratory Standards Institute. *Tubes and Additives for Venous and Capillary Blood Specimen Collection; Approved Standard—Sixth Edition*. Wayne, PA: Clinical and Laboratory Standards Institute; 2010. CLSI Document GP39‑A6.
9. Clinical and Laboratory Standards Institute. *Procedures for the Handling and Processing of Blood Specimens for Common Laboratory Tests; Approved Guideline—Fourth Edition*. Wayne, PA: Clinical and Laboratory Standards Institute; 2010. CLSI Document GP44‑A4.
10. Clinical and Laboratory Standards Institute. *Procedures for the Handling and Processing of Blood Specimens; Approved Guideline—Third Edition*. Wayne, PA: Clinical and Laboratory Standards Institute; 2004. CLSI Document H18-A3.
11. Young DS. *Effects of Preanalytical Variables on Clinical Laboratory Tests.* 3rd ed. Washington, DC: AACC Press; 2007:251.
12. Clinical and Laboratory Standards Institute. *Defining, Establishing, and Verifying Reference Intervals in the Clinical Laboratory; Approved Guideline—Third Edition*. Wayne, PA: Clinical and Laboratory Standards Institute; 2010. CLSI Document EP28‑A3c.
13.
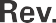

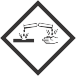

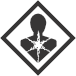
National Cholesterol Education Program. Report of expert panel on detection, evaluation, and treatment of high blood cholesterol in adults. National Heart, Lung and Blood Institute. *Arch Intern Med.* 1988;148:36-69.


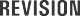

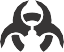

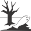


1. Clinical and Laboratory Standards Institute. *Evaluation of Detection Capability for Clinical Laboratory Measurement Procedures; Approved Guideline—Second Edition*. Wayne, PA: Clinical and Laboratory Standards Institute; 2012. CLSI Document EP17‑A2.
2. Clinical and Laboratory Standards Institute. *Evaluation of Precision of Quantitative Measurement Procedures; Approved Guideline—Third Edition*. Wayne, PA: Clinical and Laboratory Standards Institute; 2014. CLSI Document EP05‑A3.
3. Clinical and Laboratory Standards Institute. *Measurement Procedure Comparison and Bias Estimation Using Patient Samples; Approved Guideline—Third Edition*. Wayne, PA: Clinical and Laboratory Standards Institute; 2013. CLSI Document EP09‑A3.
4. Clinical and Laboratory Standards Institute. *Interference Testing in Clinical Chemistry; Approved Guideline—Second Edition*. Wayne, PA: Clinical and Laboratory Standards Institute; 2005. CLSI Document EP07‑A2.
5. Data on file at Siemens Healthcare Diagnostics.

# Definition of Symbols


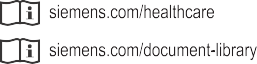
The following symbols may appear on the product labeling:

Consult instructions for use

Version of instructions for use

Internet URL address to access the electronic instructions for use

Revision

Caution

Consult instructions for use or accompanying documents for cautionary information such as warnings and precautions that cannot, for a variety of reasons, be presented on the medical device.

Biological risks

Potential biological risks are associated with the medical device.

Corrosive

Dangerous to environment

Irritant

Oral, dermal, or inhalation hazard

Inhalation hazard Respiratory or internal health

**Symbol Title and Description**

**Symbol**


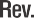

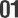


**Symbol Symbol Title and Description**

Flammable

Flammable to extremely flammable

Oxidizing


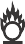


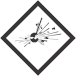
Explosive

Toxic


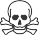


Compressed gas


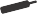


Keep away from sunlight


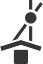


Prevent exposure to sunlight and heat.

Up

Store in an upright position.

Do not freeze


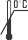


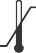

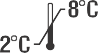
Temperature limit

Upper and lower limits of temperature indicators are adjacent to the upper and lower horizontal lines.

Handheld barcode scanner

*In vitro* diagnostic medical device


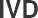


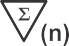
Contains sufficient for <n> tests

Total number of IVD tests the system can perform with the IVD kit reagents appears adjacent to the symbol.

Prescription device (US only)


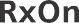


Applies only to United States-registered IVD assays.

CAUTION: Federal (USA) law restricts this device to sale by or on the order of a licensed healthcare professional.


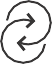
Mixing of substances Mix product before use.


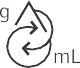
Reconstitute and mix lyophilized product before use.

Target


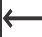

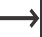
Interval


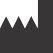

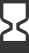

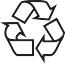

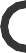

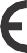


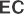

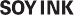

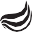

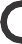

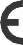

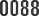

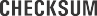

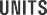

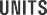


CE Mark with notified body ID number Notified body ID number can vary.

Date format (year‑month‑day)

Variable hexadecimal number that ensures the Master Curve and Calibrator defini- tion values entered are valid.

Common Units

International System of Units Material

Unique material identification number Name of control

Type of control

CE Mark

Printed with soy ink

Authorized Representative in the European Community

Use-by date

Use by the designated date. Batch code

Catalog number

Recycle

Legal Manufacturer

**Symbol Title and Description**

**Symbol**

# Legal Information


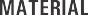

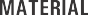

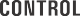

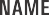

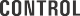

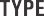


Atellica and ADVIA are trademarks of Siemens Healthcare Diagnostics. All other trademarks are the property of their respective owners.

© 2017–2020 Siemens Healthcare Diagnostics. All rights reserved.


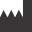
Siemens Healthcare Diagnostics Inc. 511 Benedict Avenue

Tarrytown, NY 10591 USA

[siemens.com/healthineers](http://siemens.com/healthineers)

#### Siemens Healthineers Headquarters

Siemens Healthcare GmbH Henkestr. 127

91052 Erlangen Germany

Phone: +49 9131 84-0

[siemens.com/healthineers](http://siemens.com/healthineers)
