# Supplementary material for: Soya saponins and prebiotics alter intestinal functions in Ballan wrasse (Labrus bergylta)
Source: Br J Nutr. 2023 Jan 12;130(5):765–82. doi: 10.1017/S000711452200383X (PMC10404481; doi:10.1017/S000711452200383X)
Supplement: Supplementary file 1 [file S000711452200383Xsup001.zip › S000711452200383Xsup003.docx]

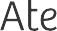

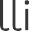

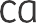

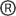

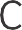


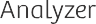
Triglycerides_2 (Trig_2)


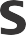

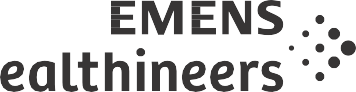


| **Current Revision and Date**a | Rev. 04, 2022-03 |
| --- | --- |
| **Product Name** | Atellica CH Triglycerides_2 (Trig_2) 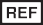 11537222 (2000 tests) |
| **Abbreviated Product Name** | Atellica CH Trig_2 |
| **Test Name/ID** | Trig_2 |
| **Systems** | Atellica CH Analyzer |
| **Materials Required but Not Provided** | Atellica CH CHEM CAL 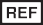 11099411 |
| **Specimen Types** | Serum, potassium EDTA plasma, lithium heparin plasma, sodium heparin plasma |
| **Sample Volume** | 5.3 µL |
| **Measuring Interval** | 15–1000 mg/dL (0.17–11.30 mmol/L) |

a A vertical bar in the page margin indicates technical content that differs from the previous version.


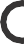

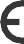


# Intended Use

The Atellica® CH Triglycerides_2 (Trig_2) assay is for *in vitro* diagnostic use in the quantitative determination of triglycerides in human serum and plasma (lithium heparin, sodium heparin, potassium EDTA) using the Atellica® CH Analyzer. Measurements obtained are used in the diagnosis and treatment of patients with diabetes mellitus, nephrosis, liver obstruction, other diseases involving lipid metabolism, or various endocrine disorders.

# Summary and Explanation

Triglycerides are water-insoluble lipids consisting of three fatty acids linked to one glycerol molecule. Triglycerides are transported in the blood as core constituents of all lipoproteins, but the greatest concentration of these molecules is carried in the triglycerides-rich chylomicrons and very low density lipoproteins (VLDL).1 Through the action of lipases and bile acids, triglycerides are hydrolyzed into glycerol and fatty acids which are absorbed by adipose tissue for storage or by other tissues requiring a source of energy. A peak concentration of chylomicron-associated triglycerides occurs within 3–6 hours after ingestion of a fat-rich meal; however, the rate of absorption of fats is highly variable, depending on the individual and dietary composition of the fat. After absorption, triglycerides are resynthesized in the epithelial cells and combined with cholesterol and a number of apolipoproteins to form chylomicrons.2

# Principles of the Procedure

The Atellica CH Trig_2 assay is based on an enzymatic procedure in which a combination of enzymes are employed for the measurement of serum or plasma triglycerides. The sample is incubated with lipoprotein lipase (LPL) enzyme reagent that converts triglycerides into free glycerol and fatty acids. Glycerol kinase (GK) catalyzes the phosphorylation of glycerol by adenosine‑5‑triphosphate (ATP) to glycerol‑3‑phosphate. Glycerol‑3‑phosphate-oxidase oxidizes glycerol‑3‑phosphate to dihydroxyacetone phosphate and hydrogen peroxide (H_2_O_2_). The catalytic action of peroxidase (POD) forms quinoneimine from H_2_O_2_, aminoantipyrine and 4‑chlorophenol. The change in absorbance due to the formation of quinoneimine is directly proportional to the total amount of glycerol and its precursors in the sample and is measured using a bichromatic (505/694 nm) endpoint technique.

## Reaction Equation

LPL

Triglycerides Glycerol + Fatty Acids

GK

Glycerol + ATP Glycerol‑3‑Phosphate + ADP

GPO

Glycerol‑3‑Phosphate + O_2_ Dihydroxyacetone phosphate + H_2_O_2_

POD

2H_2_O_2_ + Aminoantipyrine + 4‑Chlorophenol Quinoneimine + HCl + 4H_2_O

# Reagents

| **Material Description** | **Storage** | **Stabilitya** |
| --- | --- | --- |
| **Atellica CH Trig_2 Pack 1 (P1)**  Well 1 (W1)  Reagent 1 (R1)  10.8 mL  Lipoprotein lipase (22.5 kU/L); ATP (9 mmol/L); glycerol kinase  (1.5 kU/L); glycerol-3-phosphate oxidase (6.6 kU/L); 4-aminoantipyrine (2.25 mmol/L); 4-chlorophenol (18 mmol/L); peroxidase (15 kU/L); Mg2+  (67.5 mmol/L); buffer pH 7.2 (150 mmol/L); sodium azide (< 0.1%);  bovine serum albumin (0.1%)  Well 2 (W2)  Reagent 1 (R1)  10.8 mL  Lipoprotein lipase (22.5 kU/L); ATP (9 mmol/L); glycerol kinase  (1.5 kU/L); glycerol-3-phosphate oxidase (6.6 kU/L); 4-aminoantipyrine (2.25 mmol/L); 4-chlorophenol (18 mmol/L); peroxidase (15 kU/L); Mg2+  (67.5 mmol/L); buffer pH 7.2 (150 mmol/L); sodium azide (< 0.1%);  bovine serum albumin (0.1%) | Unopened at 2–8°C Onboard per well | Until expiration date on product  90 days |

a Refer to [*Storage and Stability*](#_bookmark1).

# Warnings and Precautions

For *in vitro* diagnostic use. For Professional Use.

#### CAUTION

Federal (USA) law restricts this device to sale by or on the order of a licensed healthcare professional.

Safety data sheets (SDS) available on [siemens-healthineers.com](https://siemens-healthineers.com/).

#### CAUTION

This device contains material of animal origin and should be handled as a potential carrier and transmitter of disease.

Contains sodium azide as a preservative. Sodium azide can react with copper or lead plumbing to form explosive metal azides. On disposal, flush reagents with a large volume of water to prevent buildup of azides. Disposal into drain systems must be in compliance with prevailing regulatory requirements.

Dispose of hazardous or biologically contaminated materials according to the practices of your institution. Discard all materials in a safe and acceptable manner and in compliance with prevailing regulatory requirements.

# Storage and Stability

Store reagents in an upright position, away from light. Do not freeze reagents.

For details about product material description, storage, and stability, refer to [*Reagents*](#_bookmark0).

# Onboard Stability

Discard products at the end of the onboard stability interval. For details about product onboard stability, refer to [*Reagents*](#_bookmark0).

Do not use products beyond the expiration date printed on the product labeling.

Refer to the supplementary document “Atellica Sample Handler Calibrator and QC Storage and Stability” for information about storage and stability of materials in the Cal‑QC tube storage area.

# Specimen Collection and Handling

Serum, potassium EDTA plasma, lithium heparin plasma, and sodium heparin plasma are the recommended specimen for this assay.

The handling and storage information provided here is based on data or references maintained by the manufacturer. It is the responsibility of the individual laboratory to use all available references and/or its own studies when establishing alternate stability criteria to meet specific needs.

## Collecting the Specimen

- Observe universal precautions when collecting specimens. Handle all specimens as if they are capable of transmitting disease.3
- Follow recommended procedures for collection of diagnostic blood specimens by venipuncture.4
- Follow the instructions provided with your specimen collection device for use and processing.5
- Specimens with high turbidity or particulates should be centrifuged before analysis.
- Allow blood specimens to clot completely before centrifugation.6
- Keep tubes capped at all times.6

## Storing the Specimen

Separated specimens in the primary collection device are stable for up to 7 days at 2–8°C.7

Separated specimens may be frozen for up to 30 days at ≤ -20°C.7 Do not store in a frost‑free freezer. Thoroughly mix thawed specimens and centrifuge before using.

## Transporting the Specimen

Package and label specimens for shipment in compliance with applicable federal and international regulations covering the transport of clinical specimens and etiological agents.

## Preparing the Samples

This assay requires 5.3 µL of sample for a single determination. This volume does not include the unusable volume in the sample container or the additional volume required when performing duplicates or other tests on the same sample. For information about determining the minimum required volume, refer to the system operating instructions.

Do not use samples with apparent contamination.

Remove particulates by centrifugation according to CLSI guidance and the collection device manufacturer’s recommendations.6

For a complete list of appropriate sample containers, refer to the system operating instructions.

Before placing samples on the system, ensure that samples are free of:

- Bubbles or foam.
- Fibrin or other particulate matter.

# Procedure

## Materials Provided

The following materials are provided:

| 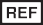 **Contents Number of Tests** |
| --- |
| 11537222 Atellica CH Trig_2 4 x 500  **Reagent 1 (R1)**  Well 1 (W1) 10.8 mL of Reagent 1  Well 2 (W2) 10.8 mL of Reagent 1 |

## Materials Required but Not Provided

The following materials are required to perform this assay, but are not provided:

| 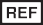 **Description** |
| --- |
| Atellica CH Analyzera |
| 11099411 Atellica CH CHEM CAL 12 x 3.0 mL calibrator  Calibrator lot-specific value sheet |
| Commercially available quality control materials |

a Additional system fluids are required to operate the system: Atellica CH Diluent, Atellica CH Wash, Atellica CH Conditioner, Atellica CH Cleaner, Atellica CH Reagent Probe Cleaner 1, Atellica CH Reagent Probe Cleaner 2, Atellica CH Reagent Probe Cleaner 4, Atellica CH Lamp Coolant, and Atellica CH Water Bath Additive. For system fluid instructions for use, refer to the Document Library.

## Assay Procedure

The system automatically performs the following steps:

1. For serum/plasma, dispenses 50 µL of primary sample and 200 µL of Atellica CH Diluent into a dilution cuvette.
2. Dispenses 35.5 µL of Reagent 1 and 59.2 µL of special reagent water into a reaction cuvette.
3. Dispenses 5.3 µL of pre‑diluted sample into a reaction cuvette.
4. Mixes and incubates the mixture at 37°C.
5. Measures the absorbance after sample addition.
6. Reports results.

**Note** For information about special reagent water, refer to the system operating instructions. Test Duration: 7.4 minutes

## Preparing the Reagents

All reagents are liquid and ready to use.

**Note** Precipitate may be observed in the wells of reagent packs. Presence of precipitate in reagent well does not impact performance characteristics of the Atellica CH Trig_2 assay.

## Preparing the System

For information about loading reagents, refer to the system operating instructions.

## Performing Calibration

For calibration of the Atellica CH Trig_2 assay, use Atellica CH CHEM CAL. Use the calibrators in accordance with the calibrator instructions for use.

## Calibration Frequency

| **Calibration Interval** | **Days** |
| --- | --- |
| Lot Calibration | 30 |
| Pack Calibration | 15 |

In addition, perform a calibration:

- - When changing lot numbers of reagents.
  - At the end of the lot calibration interval, for a specified lot of calibrated reagent on the system.
  - At the end of the pack calibration interval, for a specified lot of calibrated reagent on the system.
  - At the end of the pack calibration interval, for calibrated reagent packs on the system.
  - When indicated by quality control results.
  - After major maintenance or service.

**Note** When loading new reagents, recalibration is not required if there is a valid lot calibration. For information about the calibration interval, refer to the system operating instructions.

Follow government regulations or accreditation requirements for calibration frequency. Individual laboratory quality control programs and procedures may require more frequent calibration.

## Performing Quality Control

At least once each day of use, analyze two levels of quality control (QC) material with known triglyceride concentration. For assistance in identifying a quality control material, refer to the *Atellica CH Quality Control Material Supplement* available on [siemens-healthineers.com](https://siemens-healthineers.com/).

Additional quality control material can be used at the discretion of the laboratory. Use the quality control material in accordance with the quality control instructions for use.

In addition, perform quality control:

- Following a valid calibration.
- With use of a new lot of reagent.
- When troubleshooting test results that do not match clinical conditions or symptoms.

Follow government regulations or accreditation requirements for quality control frequency. Individual laboratory quality control programs and procedures may require more frequent quality control testing.

Acceptable performance is achieved when the analyte values obtained are within the expected control interval for the system, as indicated by the manufacturer of the control material or within the interval determined by an internal laboratory quality control procedure.

For information about entering quality control definitions, refer to the system operating instructions.

### Taking Corrective Action

If the quality control results do not fall within the expected control interval, do not report results. Perform corrective actions in accordance with established laboratory protocol. For suggested protocol, refer to the system operating instructions.

# Results

## Calculation of Results

The system determines the result using the calculation scheme described in the system operating instructions. The system reports results in mg/dL (conventional units) or mmol/L (SI units [Systèm International d’Unités]), depending on the units defined when setting up the assay.

Conversion formula: mg/dL x 0.0113 = mmol/L

For information about results outside the specified measuring interval, refer to [*Measuring*](#_bookmark2) [*Interval*](#_bookmark2).

## Interpretation of Results

Results of this assay should always be interpreted in conjunction with the patient’s medical history, clinical presentation, and other findings.

# Limitations

The following information pertains to limitations of the assay:

- - The Atellica CH Trig_2 assay is limited to the detection of triglyceride in serum, potassium EDTA plasma, lithium heparin plasma, and sodium heparin plasma.
  - As with any chemical reaction, you must be alert to the possible effect of unknown interferences from medications or endogenous substances. The laboratory and physician must evaluate all patient results in light of the total clinical status of the patient.
  - A number of substances cause physiological changes in serum or plasma analyte concentrations. A comprehensive discussion of possible interfering substances, their serum or plasma concentrations, and their possible physiological involvements is beyond the scope of this document. Consult the listed reference for specific details on known potential interfering substances.8
  - Do not use hemolyzed samples, as they may cause significant interference with this assay.
  - Venipuncture should occur prior to N-Acetyl Cysteine or Metamizole (Sulpyrine) administration due to the potential for falsely depressed results.

# Expected Values

## Reference Interval

A reference interval was established in accordance with CLSI Document EP28‑A3c 9 and verified on the Atellica CH Analyzer.9

| **Group** | **Specimen Type** | **Reference Interval mg/dL (mmol/L)** |
| --- | --- | --- |
| Healthy adults | Serum, potassium EDTA plasma, lithium heparin plasma, sodium heparin plasma10 | Normal: < 150 (< 1.70)  Borderline high: 150–199 (1.70–2.25)  High: 200–499 (2.26–5.64)  Very high: ≥ 500 (≥ 5.65) |

As with all *in vitro* diagnostic assays, each laboratory should determine its own reference interval for the diagnostic evaluation of patient results.9

# Performance Characteristics

## Measuring Interval

The Atellica CH Trig_2 assay is linear from 15 mg/dL (0.17 mmol/L) to 1000 mg/dL

(11.30 mmol/L). The system flags all values that are outside the specified measuring interval.

The lower end of the measuring interval is defined by the limit of quantitation (LoQ). Report results below the measuring interval as < 15 mg/dL (0.17 mmol/L).

## Extended Measuring Interval

An automatic repeat condition for this assay extends the measuring interval to 10,000 mg/dL (113.00 mmol/L) for serum, potassium EDTA plasma, lithium heparin plasma, and sodium heparin plasma. You may configure the system to trigger an automatic repeat. Automatic repeat results will be flagged **Autorepeat**.

## Detection Capability

The Limit of Blank (LoB) corresponds to the highest measurement result that is likely to be observed for a blank sample. The assay is designed to have an LoB ≤ limit of detection (LoD).

The LoD corresponds to the lowest concentration of triglyceride that can be detected with a probability of 95%. The assay is designed to have an LoD ≤ limit of quantitation (LoQ).

The LoQ corresponds to the lowest concentration of triglyceride in a sample at which the within-laboratory precision is ≤ 20% CV. The assay is designed to have an LoQ ≤ 15 mg/dL (0.17 mmol/L).

Detection capability was determined in accordance with CLSI Document EP17‑A2.11 The following results were obtained:

| **Specimen Type** | **Detection Capability** | **Result**  **mg/dL (mmol/L)** |
| --- | --- | --- |
| Serum/Plasma | LoB | 3 (0.03) |
|  | LoD | 6 (0.07) |
|  | LoQ | 15 (0.17) |

The LoD was determined using 360 determinations, with 180 blank and 180 low‑level replicates, and a LoB of 3 mg/dL (0.03 mmol/L).

The LoQ was determined using multiple patient samples in the interval 5–35 mg/dL (0.06–0.40 mmol/L). All samples were assayed in N=5 in each of 1 run using 3 reagent lots, over a period of 5 days.

Assay results obtained at individual laboratories may vary from the data presented.

## Precision

The assay is designed to have the following precision:

- Repeatability: CV ≤ 3.0% at 75–1000 mg/dL
- Within-Laboratory:

**–** CV ≤ 4.0% at 75–300 mg/dL

**–** CV ≤ 5.0% at 301–1000 mg/dL

Precision was determined in accordance with CLSI Document EP05‑A3.12 Samples were assayed on the Atellica CH Analyzer in duplicate in 2 runs per day for 20 days.

The following results were obtained:

| **Specimen Type** | **Na** | **Mean**  **mg/dL (mmol/L)** | **Repeatability**  **SDb**  **mg/dL (mmol/L)** | **CVc (%)** | **Within-Laboratory Precision** | |
| --- | --- | --- | --- | --- | --- | --- |
|  |  |  |  |  | **SD**  **mg/dL (mmol/L)** | **CV (%)** |
| QC1 | 80 | 144 (1.63) | 0.7 (0.01) | 0.5 | 5.7 (0.06) | 4.0 |
| Serum 1 | 80 | 398 (4.50) | 1.2 (0.01) | 0.3 | 4.6 (0.05) | 1.2 |
| Serum 2 | 80 | 693 (7.83) | 1.9 (0.02) | 0.3 | 6.6 (0.07) | 1.0 |

a Number of results.

b Standard deviation.

c Coefficient of variation.

Assay results obtained at individual laboratories may vary from the data presented.

## Reproducibility

The assay is designed to have the following reproducibility:

- - CV ≤ 8.0% at 75–300 mg/dL
  - CV ≤ 10.0% at 301–1000 mg/dL

Reproducibility was determined in accordance with CLSI Document EP05‑A3.13 Samples were assayed n=5 in 1 run for 5 days using 3 instruments and 3 reagent lots. The data were analyzed to calculate the following components of precision: repeatability, between-day, between-lot, between-instrument, and reproducibility (total). The following results were obtained:

| **Between- Repeatability Between-Day Between-Lot Instrument** | | | | | | | | | | | **Total Reproducibility** | |
| --- | --- | --- | --- | --- | --- | --- | --- | --- | --- | --- | --- | --- |
|  |  | **Mean** | **SDb** |  | **SD** |  | **SD** |  | **SD** |  | **SD** |  |
|  |  | **mg/dL** | **mg/dL** | **CVc** | **mg/dL** | **CV** | **mg/dL** | **CV** | **mg/dL** | **CV** | **mg/dL** | **CV** |
| **Sample** | **Na** | **(mmol/L)** | **(mmol/L)** | **(%)** | **(mmol/L)** | **(%)** | **(mmol/L)** | **(%)** | **(mmol/L)** | **(%)** | **(mmol/L)** | **(%)** |
| Serum QC 1 | 225 | 97  (1.10) | 0.9 (0.01) | 0.9 | 1.6 (0.02) | 1.6 | 0.6 (0.01) | 0.6 | 1.1 (0.01) | 1.2 | 2.2 (0.02) | 2.3 |
| Serum QC 2 | 225 | 138  (1.56) | 1.0 (0.01) | 0.7 | 1.7 (0.02) | 1.2 | 0.3 (0.00) | 0.2 | 1.3 (0.01) | 0.9 | 2.3 (0.03) | 1.7 |
| Serum 1 | 225 | 153  (1.73) | 1.0 (0.01) | 0.6 | 1.7 (0.02) | 1.1 | 0.0 (0.00) | 0.0 | 0.7 (0.01) | 0.5 | 2.1 (0.02) | 1.4 |
| Serum QC 3 | 225 | 209  (2.36) | 1.3 (0.01) | 0.6 | 1.6 (0.02) | 0.8 | 0.3 (0.00) | 0.2 | 1.1 (0.01) | 0.5 | 2.3 (0.03) | 1.1 |
| Serum 3 | 225 | 444  (5.02) | 1.6 (0.02) | 0.4 | 2.6 (0.03) | 0.6 | 0.5 (0.01) | 0.1 | 2.6 (0.03) | 0.6 | 4.0 (0.05) | 0.9 |
| Serum 2 | 225 | 881  (9.96) | 2.7 (0.03) | 0.3 | 5.8 (0.07) | 0.7 | 2.3 (0.03) | 0.3 | 1.2 (0.01) | 0.1 | 6.9 (0.08) | 0.8 |

a Number of results.

b Standard deviation.

c Coefficient of variation.

Assay results obtained at individual laboratories may vary from the data presented.

## Assay Comparison

The Atellica CH Trig_2 assay (y) was designed to have a correlation coefficient of ≥ 0.950 and a slope of 1.00 ± 0.10 compared to the Dimension TGL assay. Assay comparison was determined using the Deming regression model in accordance with CLSI Document EP09‑A3.14 The following results were obtained:

| **Specimen** | **Comparative Assay (x)** | **Regression Equation** | **Sample Interval** | **Na** | **rb** |
| --- | --- | --- | --- | --- | --- |
| Serum | Dimension TGL | y = 0.99x + 2 mg/dL  (y = 0.99x + 0.02 mmol/L) | 36–822 mg/dL  (0.41–9.29 mmol/L) | 102 | 0.997 |

a Number of samples tested.

b Correlation coefficient.

Agreement of the assays may vary depending on the study design, comparative assay, and sample population.

## Specimen Equivalency

Specimen equivalency was determined using the Deming linear regression model in accordance with CLSI Document EP09‑A3.14 The following results were obtained:

| **Specimen (y)** | **Reference Specimen (x)** | **Regression Equation** | **Sample Interval** | **Na** | **rb** |
| --- | --- | --- | --- | --- | --- |
| Plasma (Lithium heparin) | Serum | y = 0.99x - 8 mg/dL  (y = 0.99x - 0.09 mmol/L) | 42–713 mg/dL  (0.47–8.06 mmol/L) | 67 | 0.988 |
| Plasma (Sodium heparin) | Serum | y = 1.01x - 10 mg/dL  (y = 1.01x - 0.11 mmol/L) | 42–713 mg/dL  (0.47–8.06 mmol/L) | 67 | 0.985 |
| Plasma (Potassium EDTA) | Serum | y = 0.98x - 1 mg/dL  (y = 0.98x - 0.01 mmol/L) | 42–713 mg/dL  (0.47–8.06 mmol/L) | 67 | 0.989 |

a Number of samples tested.

b Correlation coefficient.

Agreement of the specimen types may vary depending on the study design and sample population used.

## Interferences

### Hemolysis, Icterus, and Lipemia (HIL)

Bias is the difference in the results between the control sample (does not contain the interferent) and the test sample (contains the interferent) expressed in percent. The Atellica CH Trig_2 assay is designed to have ≤ 10% interference. Bias > 10% is considered interference. Analyte results should not be corrected based on this bias.

Interference testing was performed in accordance with CLSI Document EP07‑A2.15 The following results were obtained:

| **Substance** | **Substance Concentration Conventional Units (SI Units)** | **Analyte Concentration Conventional Units (SI Units)** | **Bias**  **%** |
| --- | --- | --- | --- |
| Hemoglobin | 300 mg/dL (3.0 g/L) | 199 mg/dL (2.25 mmol/L) | 3 |
|  | 300 mg/dL (3.0 g/L) | 414 mg/dL (4.68 mmol/L) | 1 |
| Bilirubin, conjugated | 15 mg/dL (256.5 µmol/L) | 196 mg/dL (2.21 mmol/L) | -6 |
|  | 15 mg/dL (256.5 µmol/L) | 404 mg/dL (4.57 mmol/L) | -5 |
| Bilirubin, unconjugated | 5 mg/dL (85.5 µmol/L) | 200 mg/dL (2.26 mmol/L) | 3 |
|  | 5 mg/dL (85.5 µmol/L) | 416 mg/dL (4.70 mmol/L) | 0 |

Assay results obtained at individual laboratories may vary from the data presented.

### Non-Interfering Substances

The following substances do not interfere with the Atellica CH Trig_2 assay when present in serum, potassium EDTA plasma, lithium heparin plasma, and sodium heparin plasma at the concentrations indicated in the table below. Bias due to these substances is ≤ 10%.

| **Substance** | **Substance Concentration Conventional Units (SI Units)** | **Analyte Concentration Conventional Units (SI Units)** | **Bias**  **%** |
| --- | --- | --- | --- |
| Ascorbic acid | 3 mg/dL (170.3 µmol/L) | 200 mg/dL (2.26 mmol/L) | -7 |
|  | 3 mg/dL (170.3 µmol/L) | 417 mg/dL (4.71 mmol/L) | -2 |
| Etamsylate | 2 mg/dL (76.0 µmol/L) | 188 mg/dL (2.12 mmol/L) | -6 |
|  | 2 mg/dL (76.0 µmol/L) | 414 mg/dL (4.68 mmol/L) | -5 |

Assay results obtained at individual laboratories may vary from the data presented.

## Standardization

The assay is traceable to reference material SRM909 from the National Institute of Standards and Technology (NIST).

# Technical Assistance

For customer support, contact your local technical support provider or distributor. [siemens-healthineers.com](http://siemens-healthineers.com/)

# References

1. Rifai N, Warnick GR, Dominiczak MH. Handbook of Lipoprotein Testing. Washington: AACC Press, 1997, pp. 115.
2. Burtis CA, Ashwood ER. *Tietz Textbook of Clinical Chemistry*. 2nd ed. Philadelphia, PA: WB Saunders Company; 1994:1017.
3. Clinical and Laboratory Standards Institute. *Protection of Laboratory Workers From Occupationally Acquired Infections; Approved Guideline—Fourth Edition*. Wayne, PA: Clinical and Laboratory Standards Institute; 2014. CLSI Document M29‑A4.
4. Clinical and Laboratory Standards Institute. *Procedures for the Collection of Diagnostic Blood Specimens by Venipuncture; Approved Standard—Sixth Edition*. Wayne, PA: Clinical and Laboratory Standards Institute; 2007. CLSI Document GP41‑A6.
5. Clinical and Laboratory Standards Institute. *Tubes and Additives for Venous and Capillary Blood Specimen Collection; Approved Standard—Sixth Edition*. Wayne, PA: Clinical and Laboratory Standards Institute; 2010. CLSI Document GP39‑A6.
6. Clinical and Laboratory Standards Institute. *Procedures for the Handling and Processing of Blood Specimens for Common Laboratory Tests; Approved Guideline—Fourth Edition*. Wayne, PA: Clinical and Laboratory Standards Institute; 2010. CLSI Document GP44‑A4.
7. Tietz NW. *Clinical Guide to Laboratory Tests*. 3rd ed. Philadelphia, PA: WB Saunders Company; 1995:610‑611.
8. Young DS. *Effects of Preanalytical Variables on Clinical Laboratory Tests*. 5th ed. Washington, DC: AACC Press; 2000.
9. Clinical and Laboratory Standards Institute. *Defining, Establishing, and Verifying Reference Intervals in the Clinical Laboratory; Approved Guideline—Third Edition*. Wayne, PA: Clinical and Laboratory Standards Institute; 2010. CLSI Document EP28‑A3c.
10. *ATP III Guidelines At-A-Glance Quick Desk Reference*, National Cholesterol Education Program, National Institute of Health Publication No. 01-3305, May 2001.
11. Clinical and Laboratory Standards Institute. *Evaluation of Detection Capability for Clinical Laboratory Measurement Procedures; Approved Guideline—Second Edition*. Wayne, PA: Clinical and Laboratory Standards Institute; 2012. CLSI Document EP17‑A2.
12. Clinical and Laboratory Standards Institute. *Evaluation of Precision Performance of Quantitative Measurement Methods; Approved Guideline—Second Edition*. Wayne, PA: Clinical and Laboratory Standards Institute; 2004. CLSI Document EP05‑A2.
13. Clinical and Laboratory Standards Institute. *Evaluation of Precision of Quantitative Measurement Procedures; Approved Guideline—Third Edition*. Wayne, PA: Clinical and Laboratory Standards Institute; 2014. CLSI Document EP05‑A3.
14.
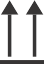
Clinical and Laboratory Standards Institute. *Measurement Procedure Comparison and Bias Estimation Using Patient Samples; Approved Guideline—Third Edition*. Wayne, PA: Clinical and Laboratory Standards Institute; 2013. CLSI Document EP09‑A3.


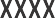

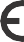

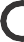

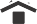

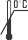

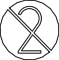

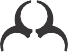

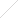

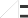


1. Clinical and Laboratory Standards Institute. *Interference Testing in Clinical Chemistry; Approved Guideline—Second Edition*. Wayne, PA: Clinical and Laboratory Standards Institute; 2005. CLSI Document EP07‑A2.

# Definition of Symbols

The following symbols may appear on the product labeling:

| **Symbol** | **Symbol Title** | **Symbol** | **Symbol Title** |
| --- | --- | --- | --- |
| 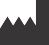 | Manufacturer | 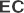 | Authorized representative in the European Community |
| 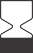 | Use-by date |  | Batch code |
|  | Catalog number | 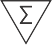 | Contains sufficient for <n> tests |
| 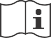 | Consult Instructions for Use | 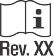 | Version of Instructions for Use |
| 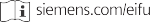 | Internet URL address to access the elec- tronic instructions for use | 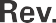 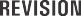 | Revision |
| 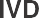 | *In vitro* diagnostic medical device | 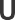 | Unique Device Identifier |
| 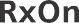 | Prescription device (US only) | 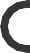 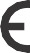 | CE Marking |
|  | CE Marking with Notified Body |  | Keep away from sunlight |
| 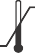 | Temperature limit | 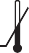 | Lower limit of temperature |
| 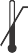 | Upper limit of temperature |  | Do not freeze |
|  | Do not re-use |  | This way up |
|  | Recycle |  | Caution |
|  | Biological risks |  | Document face upa |
| 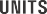 | Common Units | 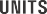 | International System of Units |
| **YYYY-MM-DD** | Date format (year-month-day) | **YYYY-MM** | Date format (year-month) |
|  | Handheld barcode scanner | 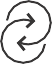 | Mixing of substances |


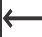

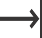


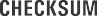

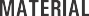

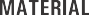

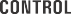

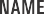

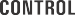

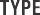


| 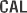 | 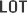 |  |
| --- | --- | --- |

a Indicates Assay-*e*Note

Target

Interval

Variable hexadecimal number that ensures the Master Curve and Calibrator definition values entered are valid.

Material

Unique material identification number

Name of control

Type of control

Calibrator lot value

Quality control lot value

**Symbol Title**

**Symbol**

**Symbol Title**

**Symbol**

# Legal Information

Atellica and Dimension are trademarks of Siemens Healthineers.

All other trademarks and brands are the property of their respective owners.

© 2021–2022 Siemens Healthineers. All rights reserved.


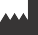
Siemens Healthcare Diagnostics Inc. 511 Benedict Avenue

Tarrytown, NY 10591 USA

**Siemens Healthineers Headquarters** Siemens Healthcare GmbH Henkestraße 127

91052 Erlangen Germany

Phone: +49 9131 84-0

[siemens-healthineers.com](https://siemens-healthineers.com/)
