# Supplementary material for: Soya saponins and prebiotics alter intestinal functions in Ballan wrasse (Labrus bergylta)
Source: Br J Nutr. 2023 Jan 12;130(5):765–82. doi: 10.1017/S000711452200383X (PMC10404481; doi:10.1017/S000711452200383X)
Supplement: Supplementary file 1 [file S000711452200383Xsup001.zip › S000711452200383Xsup004.docx]

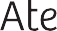

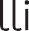

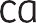

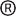

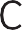


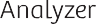
Total Protein II (TP)


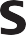

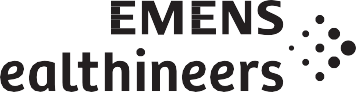


| **Current Revision and Date**a | Rev. 03, 2020-08 | |
| --- | --- | --- |
| **Product Name** | Atellica CH Total Protein II (TP) 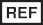 11097604 (7400 tests) | |
| **Abbreviated Product Name** | Atellica CH TP | |
| **Test Name/ID** | TP |  |
| **Systems** | Atellica CH Analyzer |  |
| **Materials Required but Not Provided** | Atellica CH CHEM CAL | 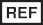 11099411 |
| **Specimen Types** | Serum, plasma (lithium heparin) |  |
| **Sample Volume** | 17.5 µL |  |
| **Measuring Interval** | 2.0–12.0 g/dL (20–120 g/L) |  |

a A vertical bar in the page margin indicates technical content that differs from the previous version.


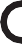

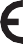


# Intended Use

The Atellica® CH Total Protein II (TP) assay is for *in vitro* diagnostic use in the quantitative determination of total protein in human serum and plasma (lithium heparin) using the Atellica® CH Analyzer. Such measurements are used in the diagnosis and treatment of a variety of diseases involving the liver, kidney, or bone marrow, as well as other metabolic and nutritional disorders.

# Summary and Explanation

The Atellica CH Total Protein II (TP) assay is based on the method of Weichselbaum using biuret reagent (cupric sulfate in an alkaline solution).1

# Principles of the Procedure

Protein peptide bonds interact with the cupric ions to form a purple complex that is measured as an endpoint reaction at 545 nm.

## Reaction Equation

OH-

Protein + CuSO4 Cuproproteinate complex

# Reagents

| **Material Description** | **Storage** | **Stabilitya** |
| --- | --- | --- |
| **Atellica CH TP Pack 1 (P1)**  Well 1 (W1)  Reagent 1 (R1)  21.7 mL  Sodium hydroxide (1.2 mol/L); Na-K-tartrate (276 mmol/L)  Well 2 (W2)  Reagent 1 (R1)  21.7 mL  Sodium hydroxide (1.2 mol/L); Na-K-tartrate (276 mmol/L)  **Pack 2 (P2)**  Well 1 (W1)  Reagent 2 (R2)  21.7 mL  Sodium hydroxide (1.2 mol/L); Na-K-tartrate (276 mmol/L); potassium iodide (180 mmol/L); cupric sulfate (72 mmol/L)  Well 2 (W2)  Reagent 2 (R2)  21.7 mL  Sodium hydroxide (1.2 mol/L); Na-K-tartrate (276 mmol/L); potassium iodide (180 mmol/L); cupric sulfate (72 mmol/L) | Unopened at 15–25°C Onboard per well | Until expiration date on product 90 days |

a Refer to [*Storage and Stability*](#_bookmark0)

## Warnings and Precautions

For *in vitro* diagnostic use. For Professional Use.

#### CAUTION

Federal (USA) law restricts this device to sale by or on the order of a licensed healthcare professional.

Safety data sheets (SDS) available on [siemens.com/healthineers](http://siemens.com/healthineers).

####
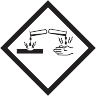
H290, H314 P280,

**P301+P330+P331, P303+P361+P353, P305+P351+P338, P310, P390, P501**

#### Danger!

May be corrosive to metals. Causes severe skin burns and eye damage. Wear protective gloves/protective clothing/eye protection/face protection. IF SWALLOWED: rinse mouth. Do NOT induce vomiting. IF ON SKIN (or hair): Remove/Take off immediately all contaminated clothing. Rinse skin with water/shower. IF IN EYES: Rinse cautiously with water for several minutes. Remove contact lenses, if present and easy to do. Continue rinsing. Immediately call a POISON CENTER or doctor/physician. Absorb spillage to prevent material damage. Dispose of contents and container in accordance with all local, regional, and national regulations.

**Contains:** Sodium hydroxide (R1)

####
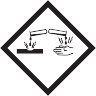
H290, H314, H412 P280, P273, P301+P330+P331, P303+P361+P353, P305+P351+P338, P310, P390, P501

**Danger!**

May be corrosive to metals. Causes severe skin burns and eye damage. Harmful to aquatic life with long lasting effects.

Wear protective gloves/protective clothing/eye protection/face protection. Avoid release to the environment. IF SWALLOWED: rinse mouth. Do NOT induce vomiting. IF ON SKIN (or hair): Remove/Take off immediately all contaminated clothing. Rinse skin with water/shower. IF IN EYES: Rinse cautiously with water for several minutes. Remove contact lenses, if present and easy to do. Continue rinsing. Immediately call a POISON CENTER or doctor/physician. Absorb spillage to prevent material damage. Dispose of contents and container in accordance with all local, regional, and national regulations.

**Contains:** Sulphuric acid copper (2+) salt (1:1), hydrate (1:5); sodium hydroxide (R2)

Dispose of hazardous or biologically contaminated materials according to the practices of your institution. Discard all materials in a safe and acceptable manner and in compliance with prevailing regulatory requirements.

**Note** For information about reagent preparation, refer to [*Preparing the Reagents*](#_bookmark2) in the

[*Procedure*](#_bookmark1) section.

## Storage and Stability

Unopened reagents are stable until the expiration date on the product when stored at 15–25°C.

Do not use products beyond the expiration date printed on the product labeling.

## Onboard Stability

Reagents are stable onboard the system for 90 days. Discard reagents at the end of the onboard stability interval. Do not use products beyond the expiration date printed on the product labeling.

# Specimen Collection and Handling

Serum and plasma (lithium heparin) are the recommended sample types for this assay.

## Collecting the Specimen

- Observe universal precautions when collecting specimens. Handle all specimens as if they are capable of transmitting disease.2
- Follow recommended procedures for collection of diagnostic blood specimens by venipuncture.3
- Follow the instructions provided with your specimen collection device for use and processing.4
- Allow blood specimens to clot completely before centrifugation.5
- Keep tubes capped at all times.5

## Storing the Specimen

Separated specimens may be stored for up to 8 hours at room temperature6 or for up to 3 days at 2–8°C7 or stored frozen for up to 180 days at -20°C.7

The handling and storage information provided here is based on data or references maintained by the manufacturer. It is the responsibility of the individual laboratory to use all available references and/or its own studies when establishing alternate stability criteria to meet specific needs.

## Transporting the Specimen

Package and label specimens for shipment in compliance with applicable federal and international regulations covering the transport of clinical specimens and etiological agents.

## Preparing the Samples

This assay requires 17.5 µL of sample for a single determination. This volume does not include the unusable volume in the sample container or the additional volume required when performing duplicates or other tests on the same sample. For information about determining the minimum required volume, refer to the online help.

**Note** Do not use specimens with apparent contamination.

Before placing samples on the system, ensure that samples are free of:

- Bubbles or foam.
- Fibrin or other particulate matter.

**Note** Remove particulates by centrifugation according to CLSI guidance and the collection device manufacturer’s recommendations.5

**Note** For a complete list of appropriate sample containers, refer to the online help.

# Procedure

## Materials Provided

The following materials are provided:

| 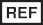 **Contents Number of Tests** |
| --- |
| 11097604 **Pack 1 (P1)** 4 x 1850  Well 1 (W1) 21.7 mL of Atellica CH TP Reagent 1 Well 2 (W2) 21.7 mL of Atellica CH TP Reagent 1  **Pack 2 (P2)**  Well 1 (W1) 21.7 mL of Atellica CH TP Reagent 2 Well 2 (W2) 21.7 mL of Atellica CH TP Reagent 2 |

## Materials Required but Not Provided

The following materials are required to perform this assay, but are not provided:

| 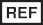 **Description** |
| --- |
| Atellica CH Analyzera |
| 11099411 Atellica CH CHEM CAL (calibrator) 12 x 3.0 mL calibrator  Calibrator lot-specific value sheet |
| Commercially available quality control materials |

a Additional system fluids are required to operate the system: Atellica CH Diluent, Atellica CH Wash, Atellica CH Conditioner, Atellica CH Cleaner, Atellica CH Reagent Probe Cleaner 1, Atellica CH Reagent Probe Cleaner 2, Atellica CH Reagent Probe Cleaner 4, Atellica CH Lamp Coolant, and Atellica CH Water Bath Additive. For system fluid instructions for use, refer to the Document Library.

## Assay Procedure

The system automatically performs the following steps:

1. For serum/plasma, dispenses 50 µL of primary sample and 200 µL of Atellica CH Diluent into a dilution cuvette.
2. Dispenses 20.8 µL of Reagent 1 and 41.7 µL of special reagent water into a reaction cuvette.
3. Dispenses 17.5 µL of pre-diluted sample into a reaction cuvette.
4. Measures the absorbance after sample addition.
5. Dispenses 20.8 µL of Reagent 2 and 41.7 µL of special reagent water into a reaction cuvette.
6. Mixes and incubates the mixture at 37°C.
7. Measures the absorbance after Reagent 2 addition.
8. Reports results.

**Note** For information about special reagent water requirements, refer to the online help. Test Duration: 10 minutes

## Preparing the Reagents

All reagents are liquid and ready to use.

## Preparing the System

Ensure that the system has sufficient reagent packs loaded in the reagent compartment. For information about loading reagent packs, refer to the online help.

## Performing Calibration

For calibration of the Atellica CH TP assay, use Atellica CH CHEM CAL. Use the calibrators in accordance with the calibrator instructions for use.

### Calibration Frequency

Perform a calibration if one or more of the following conditions exist:

- - When changing lot numbers of primary reagent packs.
  - At the end of the lot calibration interval, for a specified lot of calibrated reagent on the system.
- At the end of the pack calibration interval, for calibrated reagent packs on the system.
- When indicated by quality control results.
- After major maintenance or service, if indicated by quality control results.

At the end of the onboard stability interval, replace the reagent pack on the system with a new reagent pack. Recalibration is not required, unless the lot calibration interval is exceeded.

| **Stability Interval Days** |
| --- |
| Lot Calibration 181 |
| Pack Calibration 30 |
| Reagent Onboard Stability 90 |

For information about lot calibration and pack calibration intervals, refer to the online help. Follow government regulations or accreditation requirements for calibration frequency.

Individual laboratory quality control programs and procedures may require more frequent calibration.

## Performing Quality Control

For quality control of the Atellica CH TP assay, use at least two levels (low and high) of the appropriate quality control material of known analyte concentration. Use the quality control material in accordance with the quality control instructions for use.

For the assigned values, refer to the lot‑specific value sheet provided. A satisfactory level of performance is achieved when the analyte values obtained are within the expected control range for the system or within your range, as determined by an appropriate internal laboratory quality control scheme. Follow your laboratory’s quality control procedures if the results obtained do not fall within the acceptable limits. For information about entering quality control definitions, refer to the online help.

Follow government regulations or accreditation requirements for quality control frequency. Individual laboratory quality control programs and procedures may require more frequent quality control testing.

### Taking Corrective Action

If the quality control results do not fall within the assigned values, do not report results. Perform corrective actions in accordance with established laboratory protocol. For suggested protocol, refer to the online help.

# Results

## Calculation of Results

The system determines the result using the calculation scheme described in the online help. The system reports results in g/dL (common units) or g/L (SI units), depending on the units defined when setting up the assay.

Conversion formula: g/dL x 10 = g/L

For information about results outside the specified measuring interval, refer to [*Measuring*](#_bookmark3) [*Interval*](#_bookmark3).

## Interpretation of Results

Results of this assay should always be interpreted in conjunction with the patient’s medical history, clinical presentation, and other findings.

# Limitations

The Atellica CH TP assay is limited to the detection of total protein in human serum and plasma (lithium heparin).

Operators may see a potential interference in Atellica CH TP results for patients receiving dextran as blood volume expanders.3 This potential interference would appear as an overestimation or a positive bias in results.8

# Expected Values

## Reference Interval

A reference interval for healthy adults was established in accordance with CLSI Document EP28‑A3c and verified on the Atellica CH Analyzer.9

The reference interval for total protein is 5.7–8.2 g/dL (57–82 g/L) for adults. These data were established on the ADVIA® Chemistry system.10

As with all *in vitro* diagnostic assays, each laboratory should determine its own reference interval for the diagnostic evaluation of patient results. Consider these values as guidance only.9

# Performance Characteristics

## Measuring Interval

The Atellica CH TP assay provides results from 2.0–12.0 g/dL (20–120 g/L). The system flags all values that are outside the specified measuring interval.

## Extended Measuring Interval

An automatic repeat condition for this assay extends the measuring interval to 24.0 g/dL

(240 g/L) for serum and plasma. You may configure the system to trigger an automatic repeat. Automatic repeat results will be flagged **Autorepeat**.

## Detection Capability

Detection capability was determined in accordance with CLSI Document EP17‑A2.11 The assay is designed to have a limit of blank (LoB) ≤ limit of detection (LoD) and LoD ≤ 2.0 g/dL (20 g/L).

The LoD corresponds to the lowest concentration of total protein that can be detected with a probability of 95%. The LoD for the Atellica CH TP assay is 0.7 g/dL (7 g/L), and was determined using 120 determinations, with 60 blank and 60 low level replicates, and a LoB of 0.6 g/dL

(6 g/L).

Assay results obtained at individual laboratories may vary from the data presented.

## Precision

Precision was determined in accordance with CLSI Document EP05‑A3.12 Samples were assayed on an Atellica CH Analyzer in duplicate in 2 runs per day for 20 days (N ≥ 80 for each sample). The following results were obtained:

| **Designed to be**  **Repeatability ≤** | | | | | | **Within-Lab Pr** | **ecision** | **Designed to be**  **≤** |
| --- | --- | --- | --- | --- | --- | --- | --- | --- |
|  |  | **Mean** | **SDa** | **CVb** | **CV** | **SD** | **CV** | **CV** |
| **Sample Type** | **N** | **g/dL (g/L)** | **g/dL (g/L)** | **(%)** | **(%)** | **g/dL (g/L)** | **(%)** | **(%)** |
| Serum QC | 80 | 4.0 (40) | 0.03 (0.3) | 0.8 | 3.0 | 0.04 (0.4) | 1.0 | 6.0 |
| Serum | 80 | 7.7 (77) | 0.13 (1.3) | 1.7 | 2.5 | 0.13 (1.3) | 1.7 | 4.0 |
| Plasma | 80 | 9.6 (96) | 0.05 (0.5) | 0.5 | 2.5 | 0.13 (1.3) | 1.3 | 4.0 |

a Standard deviation.

b Coefficient of variation.

Assay results obtained at individual laboratories may vary from the data presented.

## Assay Comparison

The Atellica CH TP assay is designed to have a correlation coefficient of > 0.95 and a slope of

- 1. ± 0.1 compared to ADVIA Chemistry 1800 Total Protein II. Assay comparison was determined using the Deming linear regression model in accordance with CLSI Document EP09‑A3.13 The following results were obtained:

| **Specimen** | **Comparative Assay (x)** | **Regression Equation** | **Sample Interval** | **Na** | **rb** |
| --- | --- | --- | --- | --- | --- |
| Serum | ADVIA Chemistry 1800 Total Protein II | y = 0.98x + 0.1 g/dL (y = 0.98x + 1 g/L) | 2.8–11.7 g/dL  (28–117 g/L) | 100 | 0.997 |

a Number of samples tested.

b Correlation coefficient.

The agreement of the assay may vary depending on the study design, comparative assay, and sample population. Assay results obtained at individual laboratories may vary from the data presented.

## Specimen Equivalency

Specimen equivalency was determined using the Deming linear regression model in accordance with CLSI Document EP09‑A3.13 The following results were obtained:

| **Specimen (y)** | **Reference Specimen (x)** | **Regression Equation** | **Sample Interval** | **Na** | **rb** |
| --- | --- | --- | --- | --- | --- |
| Lithium heparin plasma | Serum | y = 0.96x + 0.3 g/dL (y = 0.96x + 3 g/L) | 2.9–11.0 g/dL  (29–110 g/L) | 64 | 0.977 |

a Number of samples tested.

b Correlation coefficient.

Agreement of the specimen types may vary depending on the study design and sample population used. Assay results obtained at individual laboratories may vary from the data presented.

## Interferences

### Hemolysis, Icterus, and Lipemia (HIL)

The Atellica CH TP assay is designed to have ≤ 10% interference from hemoglobin, bilirubin, and lipemia. Interfering substances at the levels indicated in the table below were tested in accordance with CLSI Document EP07‑A2 using the Atellica CH TP assay.14

Bias is the difference in the results between the control sample (does not contain the interferent) and the test sample (contains the interferent) expressed in percent. Bias > 10% is considered interference. Analyte results should not be corrected based on this bias.

| **Substance** | **Substance Test Concentration Common Units (SI Units)** | **Analyte Concentration g/dL (g/L)** | **Percent Bias** |
| --- | --- | --- | --- |
| Hemoglobin | 500 mg/dL (0.311 mmol/L) | 6.1 (61) | 6 |
|  | 500 mg/dL (0.311 mmol/L) | 8.1 (81) | 4 |
| Bilirubin, conjugated | 25 mg/dL (428 µmol/L) | 6.1 (61) | -1 |
|  | 25 mg/dL (428 µmol/L) | 8.0 (80) | -1 |
| Bilirubin, unconjugated | 25 mg/dL (428 µmol/L) | 5.9 (59) | 2 |
|  | 25 mg/dL (428 µmol/L) | 7.8 (78) | 1 |
| Lipemia (Intralipid®) | 500 mg/dL (5.65 mmol/L) | 6.1 (61) | -2 |
|  | 500 mg/dL (5.65 mmol/L) | 7.9 (79) | 6 |

Assay results obtained at individual laboratories may vary from the data presented.

## Standardization

The Atellica CH TP assay is traceable to a biuret reference method, which uses SRM 927 reference materials from the National Institute of Standards and Technology (NIST).

Assigned values for calibrators are traceable to this standardization.10

# Technical Assistance

For customer support, contact your local technical support provider or distributor. [siemens.com/healthineers](http://siemens.com/healthineers)

# References

- - 1. Weichselbaum TE. An accurate and rapid method for the determination of proteins in small amounts of blood serum and plasma. *Am J Clin Pathol.* 1946;10:40-49.
    2. Clinical and Laboratory Standards Institute. *Protection of Laboratory Workers from Occupationally Acquired Infections; Approved Guideline—Fourth Edition*. Wayne, PA: Clinical and Laboratory Standards Institute; 2014. CLSI Document M29‑A4.
    3. Clinical and Laboratory Standards Institute. *Procedures for the Collection of Diagnostic Blood Specimens by Venipuncture; Approved Standard—Sixth Edition*. Wayne, PA: Clinical and Laboratory Standards Institute; 2007. CLSI Document GP41‑A6.
    4. Clinical and Laboratory Standards Institute. *Tubes and Additives for Venous and Capillary Blood Specimen Collection; Approved Standard—Sixth Edition*. Wayne, PA: Clinical and Laboratory Standards Institute; 2010. CLSI Document GP39‑A6.
    5. Clinical and Laboratory Standards Institute. *Procedures for the Handling and Processing of Blood Specimens for Common Laboratory Tests; Approved Guideline—Fourth Edition*. Wayne, PA: Clinical and Laboratory Standards Institute; 2010. CLSI Document GP44‑A4.
    6.
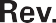

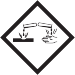
Young DS. *Effects of Preanalytical Variables on Clinical Laboratory Tests.* 3rd ed. Washington, DC: AACC Press; 2007:775-776.


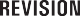

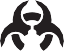

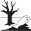


- - 1. Tietz NW. *Clinical Guide to Laboratory Tests*. 4th ed. Philadelphia: Saunders; 2006:916.
    2. Flack C, Woollen JW. Prevention of interference by dextran with biuret-type assay of serum proteins. *Clin Chem.* 1984; 30(4):559-561.
    3. Clinical and Laboratory Standards Institute. *Defining, Establishing, and Verifying Reference Intervals in the Clinical Laboratory; Approved Guideline—Third Edition*. Wayne, PA: Clinical and Laboratory Standards Institute; 2010. CLSI Document EP28‑A3c.
    4. Data on file at Siemens Healthcare Diagnostics.
    5. Clinical and Laboratory Standards Institute. *Evaluation of Detection Capability for Clinical Laboratory Measurement Procedures; Approved Guideline—Second Edition*. Wayne, PA: Clinical and Laboratory Standards Institute; 2012. CLSI Document EP17‑A2.
    6. Clinical and Laboratory Standards Institute. *Evaluation of Precision of Quantitative Measurement Procedures; Approved Guideline—Third Edition*. Wayne, PA: Clinical and Laboratory Standards Institute; 2014. CLSI Document EP05‑A3.
    7. Clinical and Laboratory Standards Institute. *Measurement Procedure Comparison and Bias Estimation Using Patient Samples; Approved Guideline—Third Edition*. Wayne, PA: Clinical and Laboratory Standards Institute; 2013. CLSI Document EP09‑A3.
    8. Clinical and Laboratory Standards Institute. *Interference Testing in Clinical Chemistry; Approved Guideline—Second Edition*. Wayne, PA: Clinical and Laboratory Standards Institute; 2005. CLSI Document EP07‑A2.

# Definition of Symbols


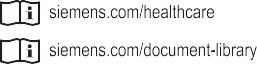
The following symbols may appear on the product labeling:

Consult instructions for use

Version of instructions for use

Internet URL address to access the electronic instructions for use

Revision

Caution

Consult instructions for use or accompanying documents for cautionary information such as warnings and precautions that cannot, for a variety of reasons, be presented on the medical device.

Biological risks

Potential biological risks are associated with the medical device.

Corrosive

Dangerous to environment

**Symbol Title and Description**

**Symbol**


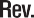

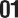


**Symbol Symbol Title and Description**

Irritant

Oral, dermal, or inhalation hazard


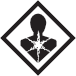
Inhalation hazard Respiratory or internal health

Flammable

Flammable to extremely flammable

Oxidizing


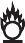


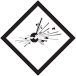
Explosive

Toxic


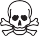


Compressed gas


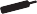


Keep away from sunlight


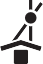


Prevent exposure to sunlight and heat.

Up

Store in an upright position.

Do not freeze


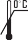


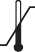

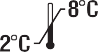
Temperature limit

Upper and lower limits of temperature indicators are adjacent to the upper and lower horizontal lines.

Handheld barcode scanner

*In vitro* diagnostic medical device


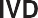


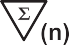
Contains sufficient for <n> tests

Total number of IVD tests the system can perform with the IVD kit reagents appears adjacent to the symbol.

Prescription device (US only)


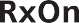


Applies only to United States-registered IVD assays.

CAUTION: Federal (USA) law restricts this device to sale by or on the order of a licensed healthcare professional.


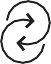
Mixing of substances Mix product before use.

**Symbol Symbol Title and Description**


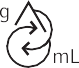
Reconstitute and mix lyophilized product before use.

Target


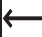

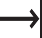
 Interval


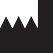
Legal Manufacturer

Authorized Representative in the European Community


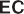


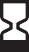
Use-by date

Use by the designated date. Batch code

Catalog number


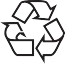
Recycle

Printed with soy ink


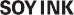

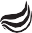


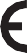

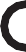
 CE Mark

CE Mark with notified body ID number Notified body ID number can vary.


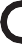

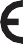

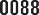


Date format (year‑month‑day)

Variable hexadecimal number that ensures the Master Curve and Calibrator defini- tion values entered are valid.


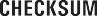


Common Units


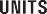


International System of Units


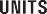


Material


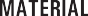


Unique material identification number


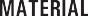


Name of control


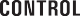

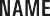


Type of control


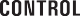

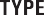


# Legal Information

Atellica and ADVIA are trademarks of Siemens Healthcare Diagnostics. All other trademarks are the property of their respective owners.

© 2017–2020 Siemens Healthcare Diagnostics. All rights reserved.


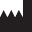
Siemens Healthcare Diagnostics Inc. 511 Benedict Avenue

Tarrytown, NY 10591 USA

[siemens.com/healthineers](http://siemens.com/healthineers)

#### Siemens Healthineers Headquarters

Siemens Healthcare GmbH Henkestr. 127

91052 Erlangen Germany

Phone: +49 9131 84-0

[siemens.com/healthineers](http://siemens.com/healthineers)
