# Supplementary material for: Soya saponins and prebiotics alter intestinal functions in Ballan wrasse (Labrus bergylta)
Source: Br J Nutr. 2023 Jan 12;130(5):765–82. doi: 10.1017/S000711452200383X (PMC10404481; doi:10.1017/S000711452200383X)
Supplement: Supplementary file 1 [file S000711452200383Xsup001.zip › S000711452200383Xsup007.docx]

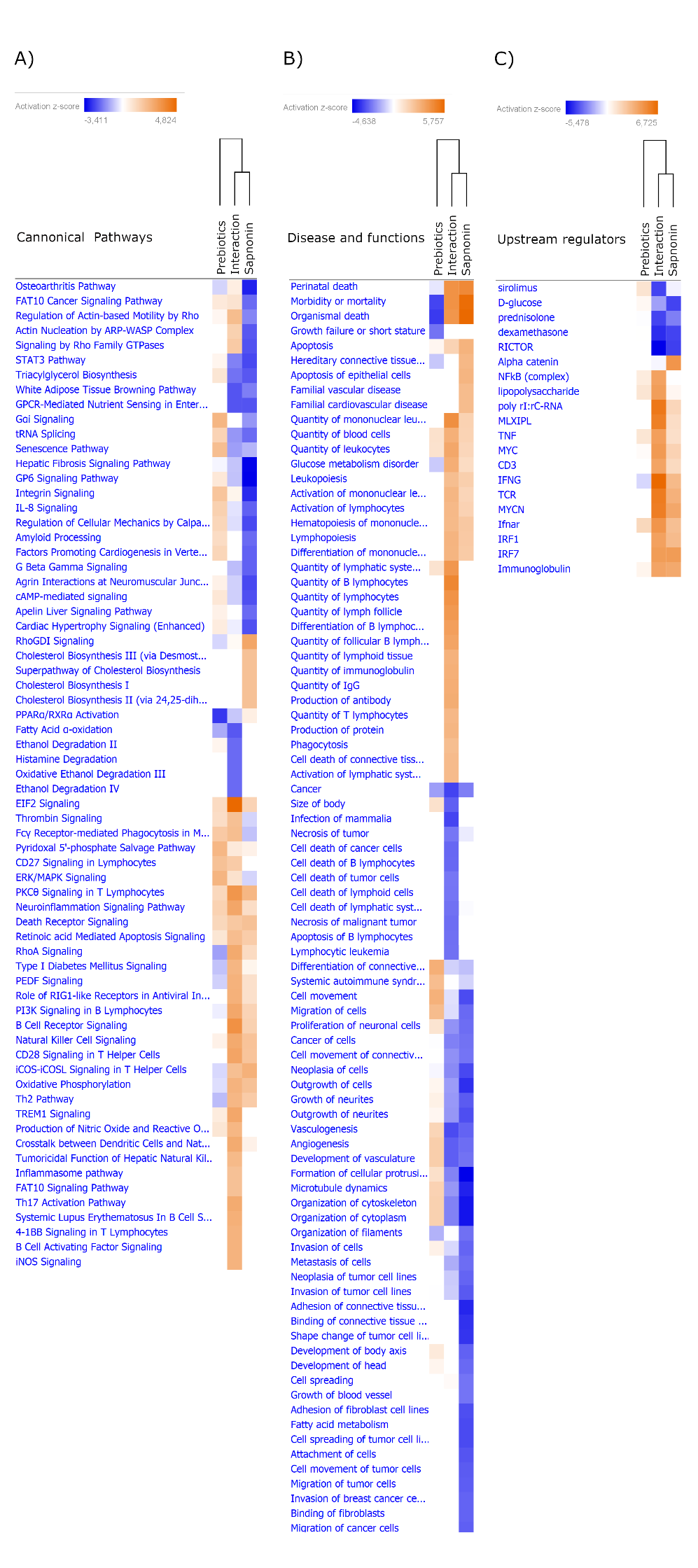


S5. Comparative Ingenuity Pathway analysis showing A) significant canonical pathways (z-score > 2) B) Disease and function (z-score > 2.5) and C) predicted upstream regulators (z-score > 4) in ballan wrasse (*Labrus bergylta*) as a result of adding either saponins, prebiotics or saponin + prebiotic in the feed. All analysis were filtered using p < 0.05 in addition to z-score filtering.
