# Supplementary material for: Soya saponins and prebiotics alter intestinal functions in Ballan wrasse (Labrus bergylta)
Source: Br J Nutr. 2023 Jan 12;130(5):765–82. doi: 10.1017/S000711452200383X (PMC10404481; doi:10.1017/S000711452200383X)
Supplement: Supplementary file 1 [file S000711452200383Xsup001.zip › S000711452200383Xsup009.docx]

# Intended use

434-91795

0318D5

The Wako NEFA-HR(2) reagent is an *in vitro* enzymatic colorimetric method assay for the quantitative determination of non-esterified fatty acids (NEFA) in serum.

# Summary and explanation of the test

Non-esterified fatty acid (NEFA) in serum binding albumin, is used as an important energy source of peripheral tissues. The amount at NEFA in serum depends on a balance between intake in liver and peripheral tissues, and the release from adipose tissues. Amount of NEFA decreases by physical exercise, increases by starvation, cold, fear or smoking. And then increase or decrease of NEFA is observed in diabetes, hepatic diseases or endocrine diseases.

NEFA had been assayed by organic solvent extraction method, which was complicated to operate. Enzymatic method using Acyl-CoA oxidase (ACOD) has become widespread due to excellent specificity and concise procedure. NEFA-HR(2) is the reagent kit for NEFA assay based on enzymatic method using 3-Methyl-N-Ethyl-N-(β-Hydroxyethyl)-Aniline (MEHA) as a violet color agent.

It gives reliable results without interference from ascorbic acid and bilirubin.

# Principle of the method

Non-esterified fatty acid (NEFA) in sample is converted to Acyl-CoA, AMP and pyrophosphoric acid (PPi) by the action of Acyl-CoA synthetase (ACS), under coexistence with coenzyme A (CoA) and adenosine 5-triphosphate disodium salt (ATP).

Obtained Acyl-CoA is oxidized and yields 2,3-trans-Enoyl-CoA and hydrogen peroxide by the action of Acyl-CoA oxidase (ACOD). In the presence of peroxidase (POD), the hydrogen per- oxide formed yields a blue purple pigment by quantitative oxidation condensation with 3-Methyl-N-Ethyl-N-(β-Hydroxyethyl)-Aniline (MEHA) and 4-aminoantipyrine (4-AA).

Non-esterified fatty acids (NEFA) concentration is obtained by measuring absorbance of the blue purple color.

# Reactions


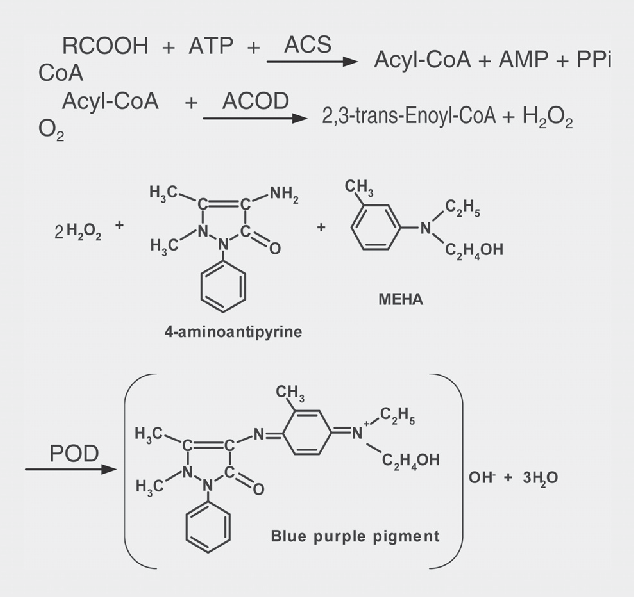


**Physical or chemical indications of instability**

The presence of precipitates in the reagents or values of control sera outside the manu- facturer’s acceptable range may be an indication of reagent’s instability.

# Instruments

The reagent is designed to be used on commercially available automated analyzers. Refer to the operating manual for a description of instrument operation and specifications. A vali- dation by the user in practice at the customer’s site in the form of measurements of adequate control or patient sera in sufficient number is indispensable.

# Reagents

## Contents and storage conditions

| **R1 Set: R2 Set:**  **Ingredients** | R1a: R1: R2a: R2: | Color A Solvent A Color B Solvent B | Store at 2–10 °C Store at 2–10 °C |
| --- | --- | --- | --- |
| **R1 Set:** |  |  |  |
| **R1a: Color A** | *(when*  ACS | *reconstituted)* | 0.53 U/ml |
|  | CoA |  | 0.31 mmol/l |
|  | ATP 4-AA |  | 4.3 mmol/l  1.5 mmol/l |

AOD 2.6 U/ml

Sodium azide 0.062 %

(Color A lyophilized) (0.8 %)

**R1: Solvent A** Phosphate Buffer, pH 7.0 50 mmol/l

Sodium azide 0.05 %

## R2 Set:

**R2a: Color B** *(when reconstituted)*

ACOD 12 U/ml

POD 14 U/ml

**R2: Solvent B** MEHA 2.4 mmol/l

# Reagent preparation

R1: Prepare R1 by mixing one bottle of Color A and Solvent A.

After preparing the R1, store at 2–10 °C and use within 1 month.

R2: Prepare R2 by mixing one bottle of Color B and Solvent B.

After preparing the R2, store at 2–10 °C and use within 1 month.

# Specimen collection and preparation

Serum can be used as specimen.

Assay samples immediately after collection, because the enzymes such as lipoprotein lipase, phospholipase etc. hydrolyze lipids and form fatty acids. Freeze sample, when a serum is stored. Stability: 2 days at 4 °C.1

In vivo heparin addition causes wrongly increased values. Because of stimulation of the lipoprotein lipase by heparin samples of patients under heparin treatment blood can be used for this determination only after appropriate pre-treatment.2

# Standard procedure

Temperature: 37 °C (Hitachi® 737)


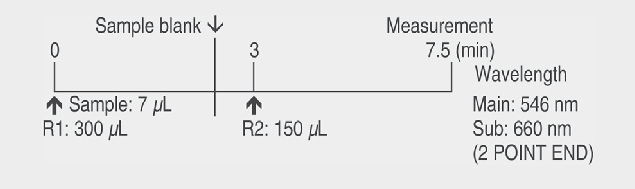


*Calibrator: Wako NEFA Standard (available separately)*

## Calculation of NEFA concentration

Calculate NEFA concentration from the calibration curve which was created from absorbance of calibrator.

*Conversion factors: mg/dL = mmol/L x 28.2 (calculated for oleic acid, MW = 282) mmol/L (mval/L = mEq/L) = mg/dL x 0.035*

## Application to the various automatic analyzers

Input the parameters according to the instructions of instruments to perform the measurement. Instrument applications are available upon request.

# Results

The final results are automatically calculated and printed in concentration. The results are given in mEq/L. Always use the same unit for the calibrator.

# Expected values3

Men: 0.1–0.60 mmol/L (2.8–16.9 mg/dL)

Women: 0.1–0.45 mmol/L (2.8–12.7 mg/dL)

Since expected values are affected by age, sex, diet, geographical location and other factors, each laboratory should establish its own expected values for this procedure.

# Performance characteristics

## Accuracy

When a control serum of known concentration is assayed, the assay value falls within the range of ± 15 % of the known concentration.

## Sensitivity

1. When purified water is assayed, the absorbance is not more than 0.140.
2. When a standard of given concentration (oleic acid 1 mEq/L) is assayed, the absorbance is 0.100–0.380.

## Precision

When a sample is assayed not less than 5 times in a run, CV of absorbance is not more than 1.5 %.

## Measurement range

0.01–4.00 mEq/l NEFA (In the case of using the standard procedure)

# Korrelation

Specimen Serum

Correlation coefficient r = 0.997 (n = 50)

Regression equation y = 1.013x - 0.043

y Wako NEFA-HR(2) (ACS – ACOD method, mEq/l)

x Wako NEFA C (ACS – ACOD method, mEq/l)

# Quality control

A quality control program is recommended for all clinical laboratories.

# References

1. Rogiers V, Stability of the long chain non-esterified fatty acid pattern in plasma and blood during different storage conditions. Clin Chim Acta. 84, 49–54 (1978).
2. Krebs, M. et al., Prevention of in Vitro Lipolysis by Tetrahydrolipstatin. Clin. Chem. 46 (7), 950–954 (2000).
3. Aufenanger, J. and Kattermann, R. Klinisch-chemische Meßgröße: Freie Fettsäuren (FFS),

S. 319–320 in Greiling / Greßner: Lehrbuch der Klinischen Chemie und Pathobiochemie, 3. edition, Schattauer (1995).

# Ordering information

## Code No. Product Package

434-91795 NEFA-HR(2) R1 Set R1a: 4 x for 50 ml

R1: 4 x 50 ml

436-91995 NEFA-HR(2) R2 Set R2a: 4 x for 25 ml

R2: 4 x 25 ml

270-77000 NEFA Standard CAL: 2 x 10 ml

# Interfering substances

1. Bilirubin gives slightly negative effect on the assay.
2. Ascorbic acid and hemolysis do not have significant effects on the assay.
3. Citrate, oxalate, EDTA and sodium fluoride do not have significant influences on the assay when they are used in their usual amounts.

# Warnings and precautions

- For *In-vitro*-diagnostic use only.
- The usage and application of this test is reserved for professional use only. Please refer to respective national and local regulations and legislation.
- Not to be used internally in humans or animals.
- Do not use the reagents described above for any purpose other than described herein. Performance cannot be guaranteed if the reagents are used in other procedures or for other purposes.
- Operate the instruments according to operator‘s manuals under appropriate conditions.
- Store the reagents under the specified conditions. Do not use reagents past the expiration date stated on each reagent container label.
- Do not use reagents which were frozen in error. Such reagents may give false results.
- After opening the reagents, it is recommended to use them immediately. When the opened reagents are stored, cap the bottles and keep them under the specified conditions.
- Do not use the containers and other materials in the package for any purposes other than those described herein.
- The vial is stoppered at reduced pressure. Slowly remove the stopper in order not to release the powder in the vial.
- When the reagent for NEFA is used at the same time as reagent for cholesterol and triglyceride, the cholesterol esterase and lipoprotein lipase in the reagent are adsorbed to cuvettes and may interfere the measured value of NEFA.
- Use NEFA Standard for calibration.
- This assay should not be used as the sole determinant for clinical diagnosis.
- If the reagents come in contact with the mouth, eyes or skin, wash off immediately with a large amount of water.
- Be careful not to cut yourself with the aluminum cap when remove it from the vial.
- When discarding the reagents, dispose of them according to local or national regu- lations. Solvent A contains 3 mg/L potassium ferrocyanide (1 mg/L as cyan).
- All the devices including reagents and reagent bottles contacted with specimen should be considered potentially infectious.
- Sodium azide may react with lead or copper plumbing to form explosive compounds. Even though the reagent contains minute quantity of sodium azide, drains should be flushed well with a large amount of water, when discarding the reagents.
